# SPOR Proteins Are Required for Functionality of Class A Penicillin-Binding Proteins in Escherichia coli

**DOI:** 10.1128/mBio.02796-20

**Published:** 2020-11-03

**Authors:** Manuel Pazos, Katharina Peters, Adrien Boes, Yalda Safaei, Calem Kenward, Nathanael A. Caveney, Cedric Laguri, Eefjan Breukink, Natalie C. J. Strynadka, Jean-Pierre Simorre, Mohammed Terrak, Waldemar Vollmer

**Affiliations:** a Centre for Bacterial Cell Biology, Biosciences Institute, Newcastle University, Newcastle upon Tyne, United Kingdom; b InBioS–Centre d'Ingénierie des Protéines, Liège University, Liège, Belgium; c Biochemistry and Molecular Biology and Centre for Blood Research, The University of British Columbia, Vancouver, British Columbia, Canada; d Department of Molecular and Cellular Physiology, Stanford University School of Medicine, Stanford, California, USA; e University of Grenoble Alpes, CNRS, CEA, IBS, Grenoble, France; f Membrane Biochemistry and Biophysics, Department of Chemistry, Faculty of Science, Utrecht University, Utrecht, The Netherlands; Nanyang Technological University

**Keywords:** SPOR domain, cell division, peptidoglycan, peptidoglycan synthases

## Abstract

Sporulation-related repeat (SPOR) domains are present in many bacterial cell envelope proteins and are known to bind peptidoglycan. Escherichia coli contains four SPOR proteins, DamX, DedD, FtsN, and RlpA, of which FtsN is essential for septal peptidoglycan synthesis. DamX and DedD may also play a role in cell division, based on mild cell division defects observed in strains lacking these SPOR domain proteins. Here, we show by nuclear magnetic resonance (NMR) spectroscopy that the periplasmic part of DedD consists of a disordered region followed by a canonical SPOR domain with a structure similar to that of the SPOR domains of FtsN, DamX, and RlpA. The absence of DamX or DedD decreases the functionality of the bifunctional transglycosylase-transpeptidase penicillin-binding protein 1B (PBP1B). DamX and DedD interact with PBP1B and stimulate its glycosyltransferase activity, and DamX also stimulates the transpeptidase activity. DedD also binds to PBP1A and stimulates its glycosyltransferase activity. Our data support a direct role of DamX and DedD in enhancing the activity of PBP1B and PBP1A, presumably during the synthesis of the cell division septum.

## Abstract (precis)

Escherichia coli has four SPOR proteins that bind peptidoglycan, of which FtsN is essential for cell division. DamX and DedD are suggested to have semiredundant functions in cell division based on genetic evidence. Here, we solved the structure of the SPOR domain of DedD, and we show that both DamX and DedD interact with and stimulate the synthetic activity of the peptidoglycan synthases PBP1A and PBP1B, suggesting that these class A PBP enzymes act in concert with peptidoglycan-binding proteins during cell division.

## INTRODUCTION

The peptidoglycan (PG) sacculus is an essential net-like polymer that surrounds the cytoplasmic membrane in most bacteria (1, 2). Although elastic, the sacculus is rigid enough to maintain the shape of a bacterial cell and protect it from bursting due to turgor. In Escherichia coli, the PG sacculus forms a thin, mostly single layer in the periplasm. PG is composed of linear glycan strands made of alternating *N*-acetylglucosamine and *N*-acetylmuramic acid residues which are connected by short stem peptides containing l- and d-amino acids (3). The glycan strands are polymerized from lipid II precursor by glycosyltransferases. The most abundant peptide cross-links connect d-Ala at position 4 of one peptide with meso-diaminopimelic acid (mDAP) at position 3 of another. These cross-links are synthesized by dd-transpeptidases called penicillin-binding proteins (PBPs), which are the primary target of β-lactam antibiotics. Most β-lactams target several PBPs, but some are more specific; for example, aztreonam selectively inhibits PBP3 (4), amdinocillin inhibits PBP2, and cefsulodin inhibits PBP1A and PBP1B (5), albeit with an 8-fold-higher affinity for PBP1A.

During the cell cycle, the PG sacculus is first enlarged during cell elongation and then split into two during cell division. Sacculus growth and division require the coordinated actions of synthetic and hydrolytic PG enzymes, while the structural integrity of the sacculus and the whole cell envelope has to be preserved at all times (6, 7). The current model suggests that dynamic multienzyme complexes, called elongasomes (or rod complexes) and divisomes, facilitate the enlargement of the sacculus during growth and cell division (8, 9). Many components of the elongasome and divisome complexes are known, but the molecular mechanisms by which these complexes function in the cell are largely unknown (6, 7).

The divisome synthesizes the cell division septum and separates the two daughter cells. The cytosolic tubulin homolog FtsZ localizes first at the future cell division site and scaffolds the recruitment of the other cell division proteins, initially by a diffusion-and-capture mechanism (10). Multiple short FtsZ filaments treadmill around the cell (Z-ring), attached to the cytoplasmic membrane by ZipA and FtsA. PBP1A and PBP1B localize to these developing division sites to insert new PG at the lateral walls in a process known as preseptal PG synthesis or PIPS (PBP3-independent PG synthesis) (11, 12). Preseptal PG synthesis takes place before septation and constriction are observed (13). Later during septation, FtsQLB and FtsN are required for the activation of septal PG synthesis (14–16), which is catalyzed by FtsW (glycosyltransferase [GTase]), PBP3 (transpeptidase [TPase]) and PBP1B or PBP1A (GTase and TPase) (17–20). Separation of the two daughter cells requires the hydrolysis of septal PG mainly by the amidases AmiA, AmiB, and AmiC, which remove stem peptides to form denuded glycan strands (21). The recruitment of EnvC (activator of AmiA and AmiB) and NlpD (activator of AmiC) to preseptal positions is essential for the temporal and spatial regulation of the amidase activity (22, 23) and therefore for correct cell constriction and separation. Lytic transglycosylases (LT) and dd-endopeptidases also contribute to septal PG hydrolysis; these cleave within the glycan strands and hydrolyze dd-cross-links, respectively (24, 25).

**FIG 1:**
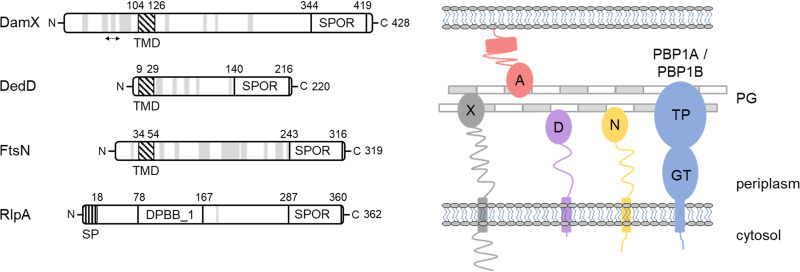
SPOR domain containing proteins in E. coli. (Left) Domains of each protein and their defining residues according to UniProt and Pfam. Potential α-helices (GOR secondary structure prediction method version IV) (53) are represented as light gray bands and the predicted coiled-coil as a double-headed arrow. TMD, transmembrane region; SPOR, SPOR domain; SP, signal peptide; DPBB_1, double-psi beta barrel; N, amino terminus; C, carboxyl terminus. (Right) Schematic representation of the SPOR proteins and the PG synthases PBP1A and PBP1B. X, DamX; A, RlpA; D, DedD; N, FtsN.

There are many nonessential proteins, often with unknown or seemingly redundant functions for sacculus growth, and these might be necessary to ensure robust growth and maintenance of the integrity of the sacculus under changing environmental conditions (26). Here, we focus on a family of proteins containing a SPOR (sporulation-related repeat) domain. These SPOR proteins bind peptidoglycan and are widely conserved among bacteria (27). Recent structural work explained their ability to bind denuded glycan strands (28). In E. coli, the SPOR proteins localize to division septa when amidases are present and show a stronger septal localization signal in mutants lacking lytic transglycosylases, supporting their binding to denuded glycan strands (29). E. coli contains four SPOR proteins, DamX, DedD, RlpA, and FtsN, of which only FtsN is essential for cell division and viability (Fig. 1) (30, 31). None of the E. coli SPOR proteins have been reported to have an enzymatic activity, but the RlpA homologue in Pseudomonas aeruginosa is an LT that acts on denuded glycan strands (32). The best-characterized SPOR protein is FtsN, which interacts with peptidoglycan (33), the cell division protein FtsA (34, 35), and the septal PG synthases PBP3 and PBP1B, stimulating both GTase and TPase synthetic activities of the latter (36). Genetic evidence supports a role for both DamX and DedD in cell division. The deletion of *damX* or *dedD* either has no detectable phenotype or causes mild cell chaining, but the lack of both genes results in a more severe cell division defect and filamentation (30, 31). The overproduction of DamX inhibits cell division and, consequently, leads to cell filamentation and death (37). DedD becomes essential in cells containing an FtsN version lacking the SPOR domain (FtsN^slm117^), a partially functional allele that supports cell viability (30). The SPOR domain of DedD is dispensable, but its transmembrane region and the adjacent periplasmic residues appear to be important for its function (38).

How DamX and DedD affect cell division and what their function is during septation are currently unknown. In this work, we provide the first genetic and biochemical evidence supporting a direct role of DamX and DedD in enhancing the activity of PBP1B and, in the case of DedD, the activity of PBP1A.

## RESULTS

### Solution-state structure of E. coli DedD.

SPOR domain protein structures have been determined for two of the four proteins in E. coli: FtsN (PDB ID 1UTA) (39) and DamX (PDB ID 2LFV) (40). Additionally, structures of the P. aeruginosa homologue of RlpA, both in the apo form and in complex with denuded glycans (PDB IDs 6I05, 6I09, 6I0N, and 6I0A) (28), and the sporulation-specific CwlC from Bacillus subtilis (PDB ID 1X60) (41) have been determined. In the context of SPOR proteins that exist in E. coli, only DedD has not been structurally characterized. We therefore decided to pursue the structure of E. coli DedD via nuclear magnetic resonance (NMR) spectroscopy to provide structural context for our functional study of the role of SPOR proteins in E. coli.

**FIG 2:**
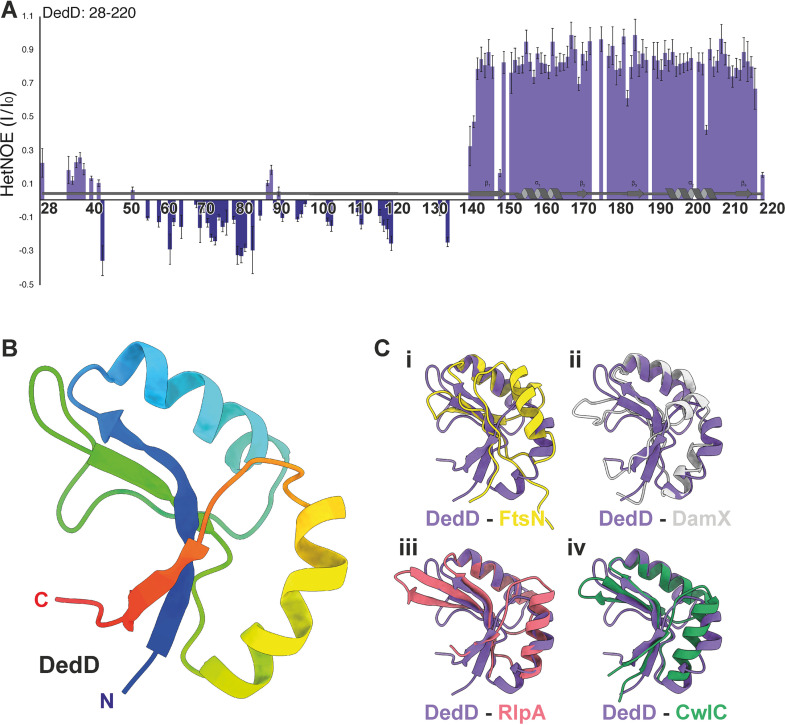
Structure of E. coli DedD. Heteronuclear ^1^H-^15^N NOE (HetNOE) ratios between saturated and reference experiments for DedD. (A) ^1^H-^15^N heteronuclear NOE of ^15^N-labeled DedD (residues 28 to 220) with secondary structuring based on the final structure. Residues within the structured region (K142 to N220) are ordered on the picosecond-to-nanosecond timescale, as indicated by an NOE value of >0.6, which is clearly differentiated from the flexible C-terminal region (D28 to K142). (B) Structured regions are depicted as a ribbon diagram with secondary structure elements shown, colored in rainbow from the N terminus (blue) to the C terminus (red). The SPOR domain regions of (i) E. coli FtsN (yellow; PDB.ID 1UTA) (39), (ii) E. coli DamX (gray; PDB.ID 2LFV) (40), (iii) P. aeruginosa RlpA (red; PDB.IDs 6I05) (28), and (iv) B. subtilis CwlC (green; PDB.ID 1X60) (41) aligned to the structured SPOR domain region of E. coli DedD (C). In each case, DedD is in purple.

An NMR-based approach was required due to the predicted combination of both structured and intrinsically disordered regions in DedD (38). The ^1^H-^15^N correlation spectrum of ^15^N-labeled DedD (residues 28 to 220 with a single transmembrane region removed) displayed both disperse peaks and intense narrow peaks with a very low ^1^H chemical shift dispersion, confirming the presence of structured and unstructured regions (Fig. 2A; Table S1 and Fig. S1, S2, and S3). ^1^H-^15^N nuclear Overhauser effect (NOE) relaxation measurements displayed positive NOE values (>0.6) for residues 144 to 220, in agreement with the presence of a globular and stable SPOR domain (Fig. 2A). Residues 36 to 141 produced low or negative NOE values, which indicate fast motion, and residues 142 and 143 showed transitional values between the structured and unstructured regions of DedD (Fig. 2A). Therefore, we confirm that the solution-state structure of DedD is that of a structured SPOR domain tethered to the inner membrane via an unstructured and flexible linking region.

10.1128/mBio.02796-20.2 TABLE S1 Structural statistics for the ensemble of 20 NMR structures of E. coli DedD. Download Table S1, DOCX file, 0.02 MB. Copyright © 2020 Pazos et al. 2020 Pazos et al. This content is distributed under the terms of the Creative Commons Attribution 4.0 International license.

10.1128/mBio.02796-20.4 FIG S1 Overlay of the 20-lowest-energy NMR structures computed for the SPOR domain of DedD depicted as a ribbon diagram with secondary structure elements shown, colored in rainbow from the N terminus (blue) to the C terminus (red). Download FIG S1, TIF file, 2.3 MB. Copyright © 2020 Pazos et al. 2020 Pazos et al. This content is distributed under the terms of the Creative Commons Attribution 4.0 International license.

10.1128/mBio.02796-20.5 FIG S2 Overlay of ^1^H-^15^N heteronuclear NOE recorded with and without initial proton saturation. The saturation experiment is light purple (positive) and dark purple (negative). The interleaved reference experiment, recorded without ^1^H saturation, is in black. Download FIG S2, PDF file, 1.1 MB. Copyright © 2020 Pazos et al. 2020 Pazos et al. This content is distributed under the terms of the Creative Commons Attribution 4.0 International license.

10.1128/mBio.02796-20.6 FIG S3 The ^1^H-^15^N BEST-TROSY spectrum of DedD 28-220 is shown, with the peak assignments indicated. Download FIG S3, TIF file, 2.7 MB. Copyright © 2020 Pazos et al. 2020 Pazos et al. This content is distributed under the terms of the Creative Commons Attribution 4.0 International license.

For the ordered and folded region of DedD, we were able to fully determine the structure. We found that residues 143 to 220 form a canonical SPOR domain consisting of a four-stranded antiparallel β-sheet flanked on one side by a pair of α-helices (Fig. 2B). On the fold level, we observe high structural similarity among all five SPOR domains that have been determined. At an atomistic level, we observe that DedD has backbone root mean square deviation (RMSD) values of 1.1, 1.4, 0.9, and 1.0 Å across 19, 17, 25, and 38 trimmed residues for FtsN, DamX, RlpA, and CwlC, respectively (Fig. 2C). This level of atomistic variability is mostly observed in the pair of α-helices that act to scaffold the β-sheet, while the β-sheet itself is more structurally conserved. This is perhaps unsurprising, as the β-sheet region was seen to form the majority of the binding interface in the liganded structures of P. aeruginosa RlpA (28). To further unravel the potential similarities and differences in the binding mode of DedD in comparison to other SPOR proteins, we generated a model of liganded DedD. In this model, we superimposed the apo NMR structure of DedD on the glycan-liganded crystal structure of RlpA (PDB ID 6I0A) (28). We see that the binding mode is very similar to that of RlpA, and as previously proposed for CwlC and FtsN models (28). This binding involves key conserved residues in the exposed basic, electropositive binding cleft (Fig. S4), such as Q147 (Q270 in RlpA), as well as analogous residues, such as L151 in place of F274 of RlpA. However, the modeled liganded DedD lacks the additional contributions from the nonconserved W365 and W416, as observed in the proposed model for DamX binding of the ligand (28). Overall, the observed conservation of the SPOR domains and structural organization suggest a shared functional overlap and interactions with other proteins involved with peptidoglycan and cell division, particularly the PG synthases.

10.1128/mBio.02796-20.7 FIG S4 Proposed DedD glycan binding. A model for denuded glycan (grey) binding by DedD was generated via superposition of the NMR structure of DedD (this work) on the liganded crystal structure of RlpA (PDB ID 6I0A) (28). Sequence conservation scores (1 to 9) calculated using the ConSurf webserver (54), are mapped onto the surface of DedD (A). Electrostatic surface potentials, calculated using APBS (55), are mapped onto the surface of DedD (B). The binding region consists mainly of a conserved, basic, electropositive region, similar to that observed in the RlpA structure. Download FIG S4, TIF file, 2.9 MB. Copyright © 2020 Pazos et al. 2020 Pazos et al. This content is distributed under the terms of the Creative Commons Attribution 4.0 International license.

### The absence of SPOR proteins increases the sensitivity to cefsulodin.

**FIG 3:**
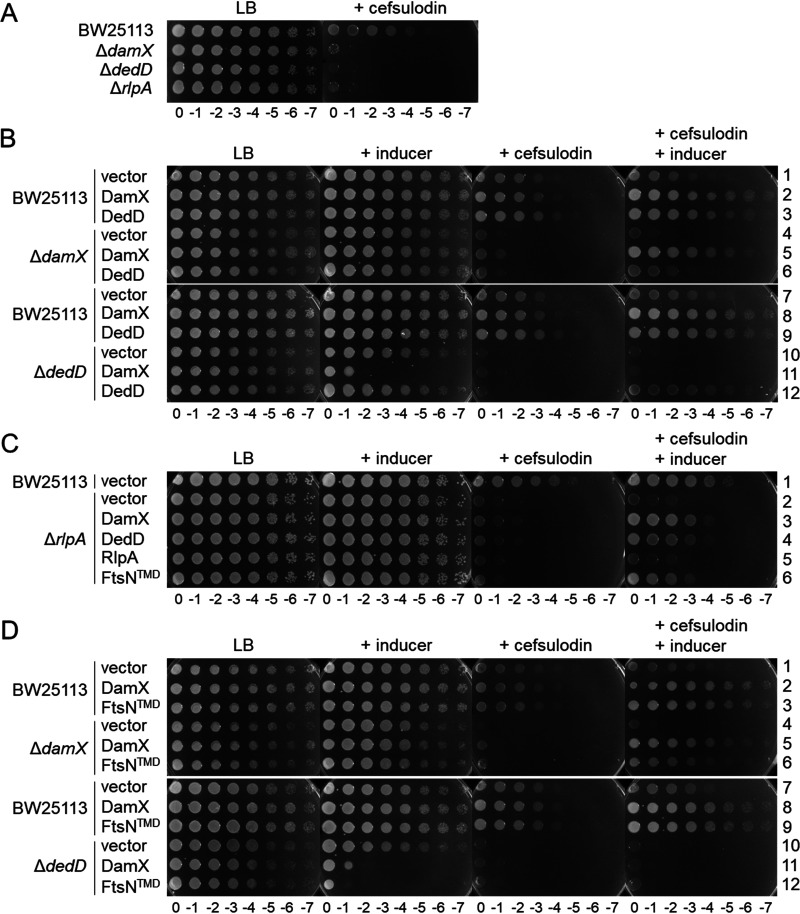
The absence of SPOR domain proteins increases the sensitivity to cefsulodin. (A) Plate spotted with tenfold serial dilutions of the indicated strains in the presence or absence of cefsulodin. (B) Overproduction of plasmid encoded DamX or DedD in wild-type, Δ*damX*, or Δ*dedD* cells in the presence or absence of cefsulodin and/or inducer. (C) Overproduction of plasmid-encoded DamX, DedD, RlpA, or DamX with the transmembrane region of FtsN (FtsN^TMD^) in wild-type or Δ*rlpA* cells in the presence or absence of cefsulodin and/or inducer. (D) Overproduction of plasmid encoded DamX or FtsN^TMD^ in wild-type, Δ*damX*, or Δ*dedD* cells in the presence or absence of cefsulodin. For all panels: cefsulodin was used at 30 mg ml^−1^, the inducer sodium salicylate was used at 10 mg ml^−1^.

We hypothesized that the SPOR proteins are required for the correct functionality of PBP1B in E. coli, based on the decrease in fitness of the single-knockout mutants when grown in the presence of cefsulodin, as revealed by a chemical genomics screening (42). Cefsulodin is a β-lactam with high affinity for PBP1A and therefore a suitable tool to assess the functionality of PBP1B, as E. coli requires at least one of these class A PBPs for viability (43). First, we confirmed the increased susceptibility to cefsulodin of the mutants with single knockouts of DamX, DedD, and RlpA (Fig. 3A), suggesting that all three proteins enhance the functionality of PBP1B in the cell. Complementation of the knockout strains by expression of the plasmid-borne genes showed that oligohistidine-tagged DamX and DedD restored the wild-type level of cefsulodin resistance (Fig. 3B, rows 5 and 12). The overexpression of the oligohistidine-tagged RlpA did not complement the mutation (Fig. 3C, row 5), suggesting that the tag interfered with the function of RlpA. A polar effect of the *rlpA* deletion on adjacent genes is unlikely, because the overexpression of DamX or DedD largely restored cefsulodin resistance (Fig. 3C, rows 3 and 4). Our further investigations focused mainly on DamX and DedD.

To study the possible redundant roles of DamX and DedD, we also tested the effect of their overexpression in wild-type cells and mutants lacking other SPOR proteins. The expression of plasmid-borne *damX* or *dedD* increased the resistance to cefsulodin in wild-type cells (Fig. 3B, rows 2, 3, 8, and 9), again supporting their positive effect on the functionality of PBP1B. In the absence of RlpA, the overproduction of DamX or DedD partially restored the resistance to cefsulodin (Fig. 3C, rows 3 and 4). DedD overproduction could partially complement the absence of *damX* (Fig. 3B, row 6), but, interestingly, DamX overproduction was lethal in Δ*dedD* cells (Fig. 3B, row 11). Together, these results suggest that both DamX and DedD are functionally semiredundant but the ratio of DamX to DedD is critical in the cell.

We then aimed to express different truncated versions of DamX to identify the region of the protein required for the observed effect on cefsulodin sensitivity (Fig. S5). However, the truncated DamX versions were unstable in the cell, and the overproduced proteins could not be detected by Western blot analysis using antibodies against the oligohistidine tag (Fig. S6). We were able to detect an overproduced DamX version containing the transmembrane region of FtsN instead of its own. Cells overproducing this DamX version enhanced the resistance to cefsulodin similarly to wild-type DamX (Fig. 3C, row 6, and Fig. 3D, rows 3 and 6; Fig. S5 and S6) and showed similar toxicity in Δ*dedD* cells (Fig. 3D, row 12).

10.1128/mBio.02796-20.8 FIG S5 DamX structural domains and constructs tested in the present work. Tenfold serial dilutions of wild type and Δ*damX* cells overproducing the above indicated plasmid encoded DamX variants were spotted on plates in the presence or absence of cefsulodin (30 μg ml^−1^). Inducer, 10 μg ml^−1^ sodium salicylate. Download FIG S5, TIF file, 2.8 MB. Copyright © 2020 Pazos et al. 2020 Pazos et al. This content is distributed under the terms of the Creative Commons Attribution 4.0 International license.

10.1128/mBio.02796-20.9 FIG S6 Overproduction of the different DamX constructs. Coomassie-stained SDS-PAGE gel (upper gel) and Western blot using anti-His tag antibody to detect the overproduction of the indicated DamX variants. Whereas the SDS-PAGE gel contained both noninduced and induced samples of each strain, the Western blot contained only the induced ones, with an empty lane between samples. The detected proteins are marked with asterisks. Download FIG S6, TIF file, 2.4 MB. Copyright © 2020 Pazos et al. 2020 Pazos et al. This content is distributed under the terms of the Creative Commons Attribution 4.0 International license.

**FIG 4:**
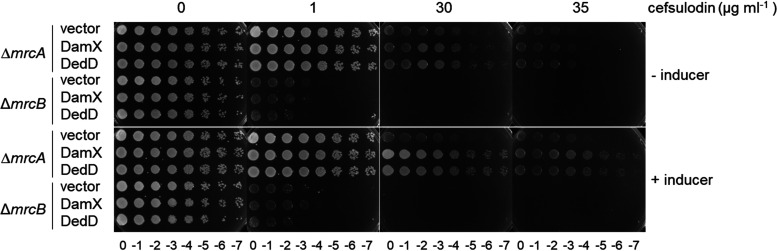
DamX and DedD enhance cellular PBP1B functionality. Tenfold serial dilutions of Δ*mrcA* or Δ*mrcB* cells overproducing the full-length plasmid-encoded DamX or DedD proteins were spotted on a plate at different cefsulodin concentrations. Inducer, 10 μg ml^−1^ sodium salicylate.

Because the cell requires at least one of the two main class A PBPs for survival, we overproduced DamX or DedD in cells lacking either PBP1A or PBP1B. We used cefsulodin at a concentration of 1 μg ml^−1^ for cells lacking PBP1B (*mrcB*) and 30 μg ml^−1^ for cells lacking PBP1A (*mrcA*). The overproduction of DamX or DedD increased the resistance to cefsulodin of cells lacking PBP1A (*mrcA*) but not of cells lacking PBP1B (*mrcB*) (Fig. 4), supporting the notion that a higher level of DamX or DedD enhances the functionality of PBP1B.

PBP1A and/or PBP1B are required to incorporate new peptidoglycan at the lateral cell wall of the future cell division site during the preseptal cell elongation phase (11). The SPOR domains of DamX and DedD are also recruited to these preseptal positions, forming ring-like structures (30). Preseptal PG synthesis is most pronounced in cells treated with aztreonam, which inhibits PBP3, the essential TPase required for the septum formation at the division site. We tested whether the MIC of aztreonam is altered when the functionality of PBP1B is reduced. Cells lacking PBP1B showed higher susceptibility to aztreonam than cells lacking PBP1A, as previously reported (44), but the absence of DamX did not alter the susceptibility to aztreonam in cells lacking PBP1A or PBP1B (Fig. S7). Together, these results show that the reduced functionality of PBP1B in the *damX* mutant manifests specifically in the presence of cefsulodin and is independent of PBP3 activity.

10.1128/mBio.02796-20.10 FIG S7 The absence of DamX does not alter the MIC of aztreonam for Δ*mrcA* or Δ*mrcB* strains. Growth inhibition of the indicated strains by concentration gradient strips of aztreonam. Download FIG S7, TIF file, 2.1 MB. Copyright © 2020 Pazos et al. 2020 Pazos et al. This content is distributed under the terms of the Creative Commons Attribution 4.0 International license.

### Interactions between SPOR proteins and PBP1A or PBP1B.

**FIG 5:**
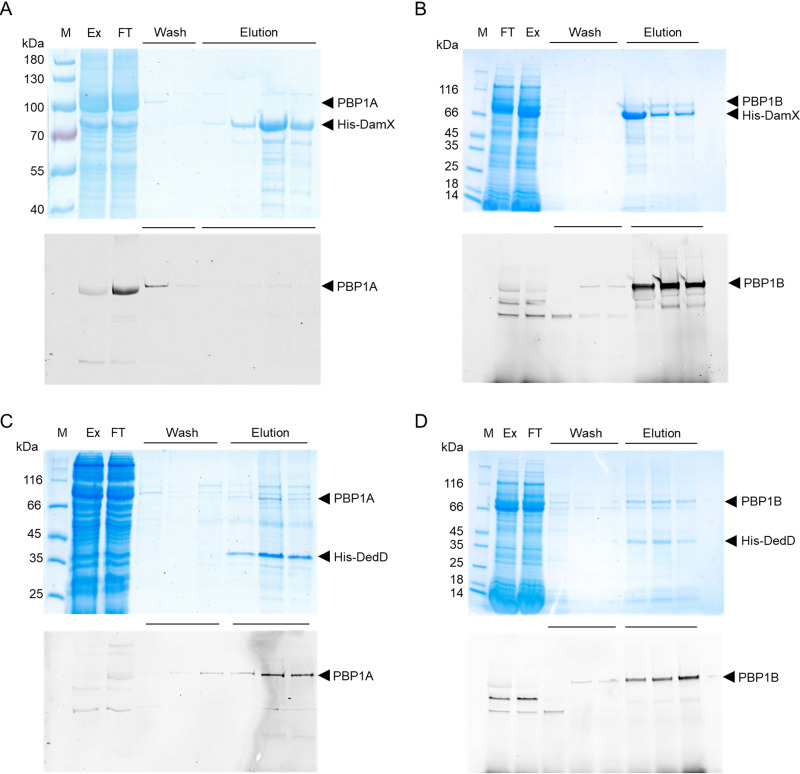
Interactions between SPOR proteins and class A PBPs. The proteins were coexpressed in E. coli and copurified on a nickel affinity column with a His tag on the SPOR protein used as bait. The PBPs were labeled using Bocillin FL followed by analysis of the samples by SDS-PAGE, fluorescence imaging (bottom), and Coomassie blue staining (top). The expressed protein pairs are indicated by arrowheads: His-DamX and PBP1A (A), His-DamX and PBP1B (B), His-DedD and PBP1A (C), and His-DedD and PBP1B (D). M, protein marker; Ex, protein extract fraction; FT, flowthrough fraction. The wash and elution fractions are indicated with horizontal lines.

To study the interaction between SPOR proteins and the class A PBPs, protein couples were coexpressed from a pETDuet plasmid into the membranes of the E. coli host, solubilized with DDM (*N*-dodecyl β-d-maltoside) detergent and copurified by affinity chromatography on a nickel-nitrilotriacetic acid (NTA) column, making use of an N-terminal His tag on the SPOR protein. The purified fractions were labeled with Bocillin FL and analyzed by sodium dodecyl sulfate-polyacrylamide gel electrophoresis (SDS-PAGE) followed by fluorescence imaging and Coomassie blue staining. The results show that PBP1B coeluted with His-DamX, while PBP1A was found only in the wash fraction (Fig. 5A and B). This suggest that DamX binds PBP1B and that the binding of DamX to PBP1A is either weak or absent. His-DedD copurified with both PBP1A and PBP1B, indicating that it interacts with both PBPs (Fig. 5C and D).

### SPOR proteins stimulate PBP1A and PBP1B.

**FIG 6:**
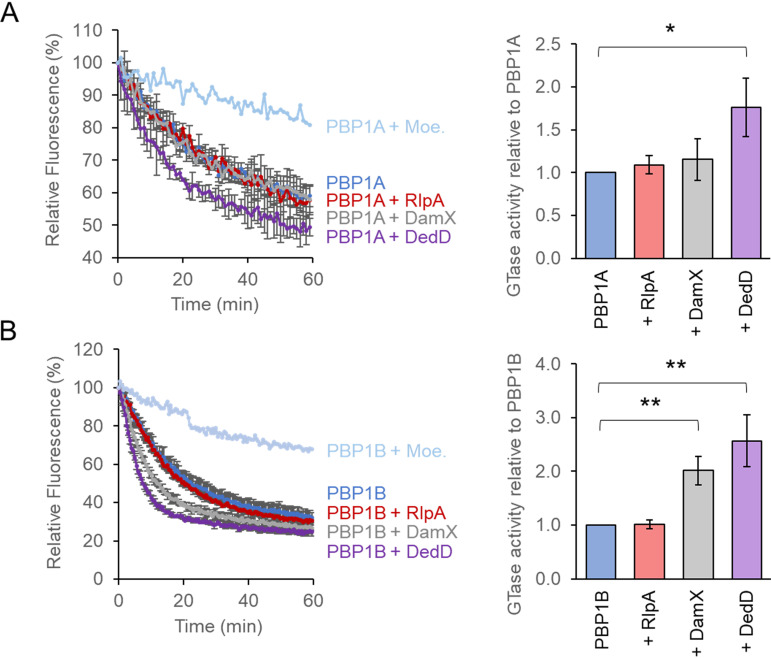
Effect of SPOR proteins on the GTase activity of PBPs. Consumption of fluorescent lipid II by the GTase activity of PBP1A (A) or PBP1B (B) in the presence of the indicated proteins. The GTase rates are shown as the decrease in fluorescence over time. Values are means and standard deviations from three independent experiments, after normalizing to the values for PBP1A or PBP1B alone. Student's *t* test (two-tailed) was used for statistical analysis (*, *P < *0.05; **, *P < *0.01). Moe., moenomycin.

We next tested if the SPOR proteins affect the GTase and TPase activities of PBP1A and PBP1B using three different *in vitro* PG synthesis assays. The first assay quantifies the consumption of fluorescent dansyl-lipid II. Both SPOR proteins had different, mild effects on the two synthases. DedD increased the GTase rate of PBP1A 1.8- ± 0.3-fold, but DamX had no effect (Fig. 6A). DedD and DamX stimulated the GTase of PBP1B to similar extents (DedD, 2.6- ± 0.5-fold; DamX, 2.0- ± 0.3-fold) (Fig. 6B). RlpA shows no effect on the GTase activity of PBP1A or PBP1B (Fig. 6A and B).

**FIG 7:**
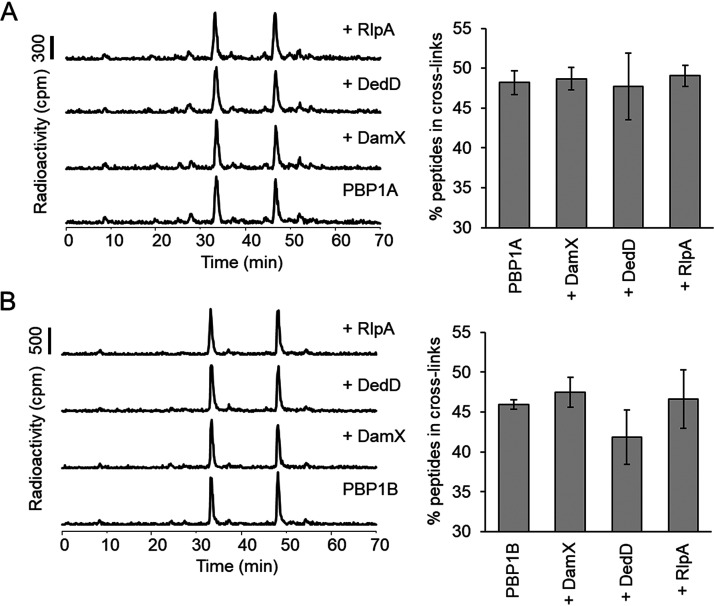
Effect of SPOR proteins on the TPase activity of PBPs. Representative HPLC chromatograms of PBP1A (A) and PBP1B (B) *in vitro* PG synthesis reactions using radioactive lipid II as the substrate in the presence of the indicated proteins. The synthesized PG was digested with cellosyl, reduced with sodium borohydride, and analyzed by HPLC. TPase activities (right) were determined by the percentage of peptides in cross-links present in the reaction products. The values are means and standard deviations from three independent experiments.

To estimate the TPase activity we quantified the percentage of cross-linked peptides present in the reaction products of an endpoint assay. Radiolabeled lipid II was used as the substrate, and the products were separated by high-pressure liquid chromatography (HPLC). We observed no significant changes in the percentage of cross-links produced by PBP1A or PBP1B (Fig. 7A and B, respectively) in the presence or absence of a SPOR protein.

**FIG 8:**
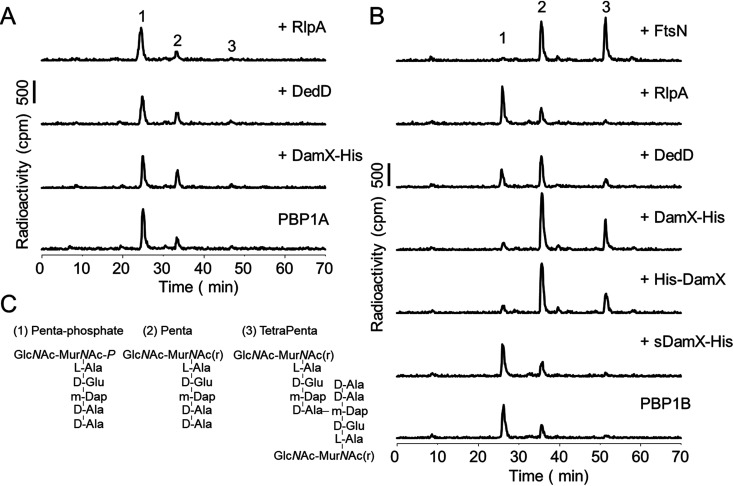
Effects of SPOR proteins on the GTase and TPase activity of PBPs at low concentrations. Representative HPLC chromatograms of PBP1A (A) and PBP1B (B) *in vitro* PG synthesis reactions using radioactive lipid II as the substrate in the presence of the indicated proteins. The synthesized PG was digested with cellosyl, reduced with sodium borohydride, and analyzed by HPLC. Peak 1 is generated from glycan strand ends and unreacted lipid II, peak 2 is a GTase product, and peak 3 is a GTase/TPase product. (C) Structures of the main products of the *in vitro* synthesis reactions.

We also tested the effect of the SPOR proteins on the activities of PBP1A and PBP1B when the synthases were present at low concentration, presumably in a less active monomeric state (19, 36). Using the HPLC-based endpoint assay, we observed no effect on the activity of PBP1A by any of the SPOR proteins (Fig. 8A). In the case of PBP1B, we observed that the addition of DedD increased the monomeric GTase product peak (Penta; compound 2) but less so the cross-linked GTase-TPase product peak (TetraPenta; compound 3) (Fig. 8B). The presence of DamX increased both Penta and TetraPenta products, resulting in an almost complete consumption of the lipid II substrate (Fig. 8B). A soluble version of DamX lacking the transmembrane region (sDamX) did not stimulate the GTase and TPase activities of PBP1B (Fig. 8B), suggesting that the transmembrane region of DamX is required for stimulation. The stimulation of PBP1B by DamX took place regardless of the N- or C-terminal position of the His tag in the purified protein. We confirmed the previously reported stimulation of PBP1B by FtsN, which was stronger than the stimulation of PBP1B by DamX, as observed by the higher consumption of the lipid II substrate (Fig. 8B, compound 1) and higher abundance of the TetraPenta product (compound 3). The structures of the main reaction products are shown in Fig. 8C.

**FIG 9:**
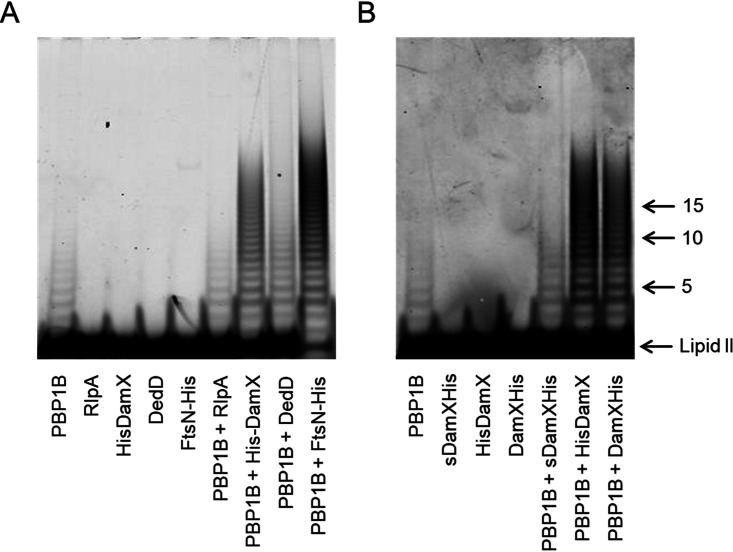
Effects of SPOR proteins on the GTase activity of PBPs at low concentrations. SDS-PAGE analysis of glycan strands synthesized by PBP1B GTase activity at low concentration in the presence of the different SPOR domain containing proteins (A) and the different DamX constructs (B). Reaction mixtures were incubated at 37°C for 1 h, using a mixture of unlabeled and ATTO 550-labeled lipid II as the substrate, in the presence of the indicated interacting proteins. The numbers refer to disaccharide units.

We also assessed the GTase activity of PBP1B using fluorescently labeled lipid II substrate, in the presence of ampicillin, and separated the produced glycan strands by SDS-PAGE. We observed an increase in the amount of glycan strand products with DamX but not RlpA or DedD (Fig. 9A). DamX with the His tag at the C terminus and DamX with the His tag at the N terminus gave similar results, but the DamX version without the transmembrane region did not stimulate PBP1B (sDamX) (Fig. 9B). Again, FtsN stimulated the GTase activity of PBP1B more strongly than DamX, consistent with our results obtained with the other assays (Fig. 9A). As expected, control reaction mixtures containing RlpA, DedD, or the different DamX proteins in the absence of PBP1B showed no GTase products (Fig. 9A and B), excluding the presence of contaminating GTases in the purified protein samples. To summarize, our activity assays showed that DamX and DedD stimulate the activity of PBP1B and, in the case of DedD, also the activity of PBP1A.

## DISCUSSION

This work identified a direct role for the SPOR proteins DamX and DedD in the function of PG synthases. The absence of DamX or DedD decreased the functionality of PBP1B in the cell, leading to an increase in the susceptibility to cefsulodin, which primarily inhibits PBP1A. We present the structure of the SPOR domain of DedD and modeled its binding to glycan chains. Our biochemical data highlight the connection between PG synthases and PG-binding proteins during cell division. Presumably, this connection contributes to stabilizing the inward-growing septal PG layer. The decreased functionality of PBP1B in the *dedD* and *damX* mutants was reverted by the ectopic expression of the respective genes from a plasmid, excluding a polar effect. Our cellular viability data support a semiredundant role for DamX and DedD in enhancing the functionality of PBP1B, and the effect is also seen in wild-type cells. An explanation for these effects might come from our biochemical data, which show that DedD and DamX both have a small but significant stimulatory effect on the GTase and TPase activities of PBP1B.

The previously reported DamX-associated phenotypes are all linked to cell division. A strong overproduction of DamX leads to cell filamentation and death (37). The additional deletion of chromosomal *damX* in the background of a *dedD* mutant causes severe filamentation (30), which can be reversed by mild overproduction of DamX (31). Our results show that mild overproduction of DamX improves the viability of cefsulodin-challenged cells with the exception of Δ*dedD* cells, in which the mild overproduction of DamX was toxic. However, DedD overproduction improved the viability of all strains in the presence of cefsulodin and was not toxic in Δ*damX* cells. These results suggest that an optimal ratio of DedD to DamX is important to maintain cell viability and that DedD is capable of “neutralizing” the toxicity of increased DamX levels. Presumably, DamX and DedD have semiredundant roles in the stimulation of class A PBPs despite having otherwise different, but complementary, roles during cell division. Both proteins interact with FtsQ in a bacterial two-hybrid assay (30, 31), and the absence of DamX improves the viability of *ftsQ*(ts) cells under nonpermissive conditions, suggesting that DamX antagonizes FtsQ function (31). Perhaps DedD protects FtsQ from being antagonized by DamX at the division site, explaining why a mild increase of DamX is toxic only when DedD is absent. In our activity assays, DedD slightly stimulated not only PBP1B but also the GTase of PBP1A, which might explain the more severe cell division defects in the absence of DedD than in the absence of DamX. Because the absence of DedD is better tolerated in cells lacking PBP1A than PBP1B (38) and the simultaneous lack of PBP1A and PBP1B is lethal (43), we hypothesize that DedD is more specific for the function of PBP1A and DamX is more specific for that of PBP1B.

All SPOR proteins localize at the cell division site in E. coli, but the timing seems to be different for each of them. In case of FtsN, a small fraction of the total cellular protein amount is recruited to the division site by an interaction with FtsA before septum synthesis begins (35). FtsN-FtsA connect the cytosolic FtsZ ring with PBP1A and PBP1B to direct preseptal PG synthesis (12). Despite their similar structures (Fig. 2), their SPOR domains also show different localization patterns. Unlike the SPOR domains of FtsN and RlpA, those of DamX and DedD are able to form ring-like structures in the absence of constriction (30).

The localization of DedD at the division site and its functionality are significantly diminished but not abolished in the absence of its SPOR domain, which renders the N-terminal transmembrane and adjacent residues essential (38). Since mutations in the transmembrane region abolish the recruitment of a SPOR less DedD to the division site (38), it is tempting to speculate that the interaction of DedD with the class A PBPs takes place through the transmembrane regions of interacting proteins and that such interaction contributes to the recruitment of DedD to the division site and to the regulation of septation.

DamX contains a large and likely folded cytoplasmic domain, together with the largest periplasmic linker region of all the inner membrane SPOR proteins. DamX localizes at the division sites in an FtsZ-dependent and FtsA-, FtsQ-, PBP3-, or FtsN-independent manner (31). The spatial and temporal pattern of DamX localization, together with its positive effect on PBP1B, is consistent with a role during the synthesis of preseptal PG. The SPOR domain is needed to efficiently target DamX to the division site and to cause cell division inhibition when DamX is overproduced (40). Together, these results suggest that DedD and DamX might recognize and bind to specific structures in PG present at the division site before the septum synthesis starts, which could be either denuded glycan strands (28) or perhaps the 1,6-anhydro ends of glycan strands (33, 36). Later, during septation, SPOR proteins provide a connection between PG synthases and the inward-growing septal PG, which may function to stabilize the constricting cell envelope and/or regulate PG synthesis. It remains to be seen in future work how the processive synthesis of septal PG is regulated by interactions with PG-binding proteins.

## MATERIALS AND METHODS

### Strains, plasmids, and growth conditions.

E. coli strains and plasmids used are listed in Table S2. Primers and methods used to construct the strains and plasmids are detailed in Text S1.

10.1128/mBio.02796-20.1 TEXT S1 Methods for the preparation of strains and plasmids. Download Text S1, DOCX file, 0.02 MB. Copyright © 2020 Pazos et al. 2020 Pazos et al. This content is distributed under the terms of the Creative Commons Attribution 4.0 International license.

10.1128/mBio.02796-20.3 TABLE S2 Strains and plasmids. Download Table S2, DOCX file, 0.02 MB. Copyright © 2020 Pazos et al. 2020 Pazos et al. This content is distributed under the terms of the Creative Commons Attribution 4.0 International license.

Unless stated otherwise, E. coli cells were grown in Miller Luria-Bertani (LB) medium (1% tryptone, 0.5% yeast extract, 1% NaCl) for protein production. When appropriate, antibiotics were added to the medium (20 μg ml^−1^ chloramphenicol, 50 μg ml^−1^ kanamycin, 100 μg ml^−1^ ampicillin).

### Protein purification.

The following proteins were purified following published protocols: PBP1A (20), PBP1B (19), and FtsN-His (45).

**(i) RlpA.** LOBSTR (low-background strain) E. coli cells containing plasmid pPZW25 were grown in 2 liters of LB medium supplemented with kanamycin at 37°C to an optical density at 578 nm (OD_578_) of 0.4 to 0.5. Protein overproduction was induced by addition of 0.5 mM IPTG (isopropyl-β-d-thiogalactopyranoside) to the cell culture, which was further incubated for 4 h at 37°C. Cells were harvested by centrifugation (6,200 × *g*, 15 min, 4°C), and the pellet was resuspended in buffer I (25 mM Tris-HCl, 1 M NaCl [pH 7.5]). After addition of 200 μM phenylmethylsulfonylfluoride (PMSF), a 1-in-1,000 dilution of protease inhibitor cocktail (Sigma-Aldrich) and DNase, the cells were disrupted by sonication (Branson Digital). The cell lysate was centrifuged (130,000 × *g*, 60 min, 4°C), and the supernatant was incubated overnight with 4 ml of nickel-nitrilotriacetic acid (Ni-NTA) Superflow (Qiagen), which had been pre-equilibrated in buffer I containing 20 mM imidazole, at 4°C with gentle stirring. The resin was poured into a gravity column and washed with 25 volumes of wash buffer (25 mM Tris-HCl, 1 M NaCl, 10 mM MgCl_2_, 20 mM imidazole [pH 7.5]). Bound protein was eluted with elution buffer (25 mM Tris-HCl, 1 M NaCl, 10 mM MgCl_2_, 400 mM imidazole [pH 7.5]). The Ni-NTA-eluted protein was dialyzed against 1 liter of dialysis buffer I (25 mM Tris-HCl, 500 mM NaCl, 10 mM MgCl_2_ [pH 7.5]) for 30 min; 500 ml of dialysis buffer I was replaced with 500 ml dialysis buffer II (25 mM Tris-HCl, 300 mM NaCl, 10 mM MgCl_2_ [pH 7.5]) and further dialyzed for 30 min. Restriction-grade thrombin (4 U ml^−1^; Merck Millipore, Darmstadt, Germany) was added to remove the oligohistidine tag during overnight dialysis against 1 liter of dialysis buffer II at 4°C. The sample was diluted 1:1 with buffer no salt (25 mM Tris-HCl, 10 mM MgCl_2_ [pH 7.5]) and applied in AKTA A buffer (25 mM Tris-HCl, 10 mM MgCl_2_, 150 mM NaCl [pH 7.5]) to a 5-ml HiTrap Q HP column using an ÄKTA Prime system (GE Healthcare Bio-Sciences) for anion-exchange chromatography (flow rate, 1 ml min^−1^). Although a gradient from 150 mM to 1 M NaCl was applied, the protein was present in the flowthrough and wash. The protein was dialyzed against 3 liters of storage buffer (25 mM HEPES-NaOH, 500 mM NaCl, 10 mM MgCl_2_, 10% glycerol [pH 7.5]) and stored at −80°C.

**(ii) His-DamX.** LOBSTR cells containing plasmid pPZW23 were grown in 2 liters of LB medium supplemented with kanamycin at 37°C to an OD_578_ of 0.4 to 0.5. Protein overproduction was induced by addition of 0.5 mM IPTG to the cell culture, which was further incubated for 4 h at 37°C. Cells were harvested by centrifugation (6,200 × *g*, 15 min, 4°C), and the pellet was resuspended in buffer I (25 mM Tris-HCl, 1 M NaCl [pH 7.5]). After addition of 200 μM PMSF, a 1-in-1,000 dilution of protease inhibitor cocktail (Sigma-Aldrich) and DNase, the cells were disrupted by sonication (Branson Digital), and the cell lysate was centrifuged (130,000 × *g*, 60 min, 4°C). The supernatant was discarded, and the membrane pellet was resuspended in extraction buffer (25 mM Tris-HCl, 1 M NaCl, 2% Triton X-100 reduced, 10 mM MgCl_2_, 10% glycerol [pH 7.5]) and incubated overnight with mixing at 4°C. Resuspended sample was centrifuged (130,000 × *g*, 60 min, 4°C), and the supernatant was incubated with Ni-NTA Superflow (Qiagen) as described for RlpA. The protein did not bind to the resin and was collected from the flowthrough. The flowthrough containing the protein was dialyzed against 3 liters of dialysis buffer I (25 mM Tris-HCl, 150 mM NaCl, 10 mM MgCl_2_ [pH 7.5]) overnight. The sample was applied in AKTA A buffer (25 mM Tris-HCl, 10 mM MgCl_2_, 150 mM NaCl, 0.2% Triton X-100 reduced [pH 7.5]) to a 5-ml HiTrap Q HP column using an ÄKTA Prime system (GE Healthcare Bio-Sciences), and the protein was collected in the flowthrough. The protein was dialyzed against 3 liters of dialysis buffer II (10 mM sodium acetate, 150 mM NaCl, 10 mM MgCl_2_ [pH 4.8]) overnight. The sample was applied in dialysis buffer II containing 0.2% Triton X-100 reduced to a 5-ml HiTrap SP HP column using an ÄKTA Prime system (GE Healthcare Bio-Sciences) for cation-exchange chromatography (flow rate, 1 ml min^−1^). The protein was eluted using a gradient from 150 mM to 2 M NaCl. Protein-containing fractions were dialyzed against 3 liters of storage buffer (25 mM HEPES NaOH, 10 mM MgCl_2_, 150 mM NaCl, 10% glycerol [pH 7.5]) and stored at −80°C.

**(iii) DamX-His and sDamX-His.** LOBSTR cells containing plasmid pPZW26 and pPZW27 were grown in 2 liters of LB medium supplemented with kanamycin at 37°C to an OD_578_ of 0.4 to 0.5. Protein overproduction was induced by addition of 0.5 mM IPTG to the cell culture which was further incubated for 4 h at 37°C. Cells were harvested by centrifugation (6,200 × *g*, 15 min, 4°C), and the pellet was resuspended in buffer I (25 mM Tris-HCl, 1 M NaCl [pH 7.5]). After addition of 200 μM PMSF, a 1-in-1,000 dilution of protease inhibitor cocktail (Sigma-Aldrich), and DNase, the cells were disrupted by sonication (Branson Digital), and the cell lysate was centrifuged (130,000 × *g*, 60 min, 4°C). For DamX-His, the supernatant was discarded, and the membrane pellet was resuspended in extraction buffer (25 mM Tris-HCl, 1 M NaCl, 2% Triton X-100 reduced, 10 mM MgCl_2_, 10% glycerol [pH 7.5]) and incubated overnight with mixing at 4°C. Resuspended sample was centrifuged (130,000 × *g*, 60 min, 4°C), and the supernatant was incubated with Ni-NTA Superflow (Qiagen), washed, and eluted as described for RlpA using buffers containing 0.2% Triton X-100 reduced. The eluted protein was dialyzed against 3 liters of storage buffer (25 mM HEPES NaOH, 10 mM MgCl_2_, 150 mM NaCl, 10% glycerol [pH 7.5]) and stored at −80°C. sDamX-His was purified from the supernatant of the cell lysate centrifugation using the protocol for DamX-His but omitting detergent in buffers. The eluted protein was dialyzed against 3 liters of dialysis buffer I (25 mM Tris-HCl, 200 mM NaCl, 10 mM MgCl_2_ [pH 7.5]) for 1.5 h and against 3 liters of dialysis buffer II (10 mM sodium acetate, 200 mM NaCl, 10 mM MgCl_2_ [pH 4.8]) overnight. The sample was diluted 1:1 with buffer no salt (10 mM sodium acetate, 10 mM MgCl_2_ [pH 4.8]) and applied in AKTA A buffer (10 mM sodium acetate, 100 mM NaCl, 10 mM MgCl_2_ [pH 4.8]) to a 5-ml HiTrap SP HP column using an ÄKTA Prime system (GE Healthcare Bio-Sciences) for cation-exchange chromatography (flow rate, 1 ml min^−1^). The protein was eluted in a gradient from 100 mM to 2 M NaCl. Protein-containing fractions were dialyzed against storage buffer (25 mM HEPES NaOH, 150 mM NaCl, 10 mM MgCl_2_, 10% glycerol [pH 7.5]) and stored at −80°C.

**(iv) DedD.** LOBSTR cells containing plasmid pPZW24 were grown in 2 liters of LB medium supplemented with kanamycin at 37°C to an OD_578_ of 0.4 to 0.5. Protein overproduction was induced by addition of 0.5 mM IPTG to the cell culture, which was further incubated for 4 h at 37°C. Cells were harvested by centrifugation (6,200 × *g*, 15 min, 4°C) and the pellet was resuspended in buffer I (25 mM Tris-HCl, 1 M NaCl [pH 7.5]). After addition of 200 μM PMSF, a 1-in-1,000 dilution of protease inhibitor cocktail (Sigma-Aldrich) and DNase, the cells were disrupted by sonication (Branson Digital), and the cell lysate was centrifuged (130,000 × *g*, 60 min, 4°C). The supernatant was discarded, and the membrane pellet was resuspended in extraction buffer (25 mM Tris-HCl, 1 M NaCl, 2% Triton X-100 reduced, 10 mM MgCl_2_, 10% glycerol [pH 7.5]) and incubated overnight with mixing at 4°C. Resuspended sample was centrifuged (130,000 × *g*, 60 min, 4°C), and the supernatant was incubated with Ni-NTA Superflow (Qiagen), washed, and eluted as described for RlpA using buffers containing 0.2% Triton X-100 reduced. Restriction-grade thrombin (4 U ml^−1^; Merck Millipore) was added to the Ni-NTA-eluted protein to remove the oligohistidine tag during dialysis against 3 liters of dialysis buffer I (25 mM Tris-HCl, 1 M NaCl, 10 mM MgCl_2_ [pH 7.5]) for 20 h at 4°C. Sample was dialyzed against 3 liters of dialysis buffer II (10 mM sodium acetate, 500 mM NaCl, 10 mM MgCl_2_ [pH 4.8]) for 4 h at 4°C and 3 liters of dialysis buffer III (10 mM sodium acetate, 300 mM NaCl, 10 mM MgCl_2_ [pH 4.8]) for 18 h at 4°C. The sample was diluted 1:1 with no-salt buffer (10 mM sodium acetate, 10 mM MgCl_2_, 0.2% Triton X-100 reduced [pH 4.8]) and applied in AKTA A buffer (10 mM sodium acetate, 150 mM NaCl, 10 mM MgCl_2_, 0.2% Triton X-100 reduced [pH 4.8]) to a 5-ml HiTrap SP HP column using an ÄKTA Prime system (GE Healthcare Bio-Sciences) for cation-exchange chromatography (flow rate, 1 ml min^−1^). The protein eluted in a gradient from 150 mM to 2 M NaCl. Protein-containing fractions were dialyzed against storage buffer (25 mM HEPES NaOH, 150 mM NaCl, 10 mM MgCl_2_, 10% glycerol [pH 7.5]) and stored at −80°C.

### Protein coexpression, copurification, and Bocillin FL labeling.

C43(DE3) cells transformed with pETDuet plasmids (Table S2) were grown in 500 ml Miller Luria-Bertani (LB) supplemented with ampicillin (100 μg ml^−1^) at 37°C to an *A*_600_ of 0.8. Protein expression was induced for 3.5 h by addition of 0.5 mM IPTG. Cells were collected by centrifugation at 4,000 × *g* for 20 min at 15°C and resuspended in a buffer containing 20 mM Tris-HCl (pH 8.0), 300 mM NaCl, and EDTA-free protease inhibitor cocktail (Roche). The cells were lysed by three passages through a cell homogenizer (Emulsiflex C3; Avestin). After centrifugation at 4,000 × *g* for 20 min at 4°C, the supernatant was recovered and spun down at 150,000 × *g* for 1 h at 4°C. The pelleted membranes were solubilized in 25 mM Tris-HCl (pH 8.0), 500 mM NaCl, 10% (vol/vol) glycerol, 40 mM DDM (Inalco) and 1 tablet of complete EDTA-free protease inhibitors (Sigma) per 50 ml of buffer. The mixture was incubated for 1 h at room temperature under gentle agitation followed by centrifugation at 150,000 × *g* for 1 h at 4°C. The supernatant containing the solubilized membrane proteins was loaded onto a HisTrap column (GE HealthCare) conditioned in buffer B (25 mM Tris-HCl [pH 7.5], 500 mM NaCl, 4 mM DDM). After a wash with buffer B supplemented with 80 mM imidazole, the proteins were eluted in 0.5- to 1-ml fractions using a linear gradient of imidazole from 80 to 500 mM. A 15-μl portion of each fraction was incubated with 2 μM Bocillin FL for 30 min at 37°C. The fractions were analyzed by SDS-PAGE, followed by fluorescence imaging and Coomassie blue staining.

### Protein expression for NMR.

For labeled DedD (residues 28 to 220) purification, pYS001 was transformed into BL21(DE3). This strain was cultured in 1 liter of M9 containing 1 g/liter of ammonium chloride and 2 g/liter of glucose at 37°C to an OD_600_ of 0.6, at which point 1 mM IPTG was added to induce protein overproduction overnight at 25°C. Harvested cell pellets were resuspended in lysis buffer (20 mM HEPES [pH 8.0], 300 mM NaCl, 10% glycerol) and lysed by processing twice with a homogenizer (15 kPa; Avestin). Cellular debris was pelleted by centrifugation at 125,000 × *g* for 1 h. The resultant supernatant was loaded onto 10 ml Ni^2+^-saturated Ni-NTA Superflow beads (Qiagen) and washed with 65 mM imidazole in 20 mM HEPES (pH 8.0)–300 mM NaCl, and the protein was eluted with 300 mM imidazole in the previous buffer. Fractions containing pure DedD were pooled and desalted into a buffer of 20 mM HEPES (pH 8.0) and 300 mM NaCl. Protein was frozen rapidly in liquid nitrogen and stored at −80°C until required.

### Spot plate assay.

Cells were grown overnight at 30°C, the optical density was normalized for each strain assayed in the plate, and the cells were spotted in a 10-fold dilution series on Lennox LB plates (1% tryptone, 0.5% yeast extract, 0.5% NaCl), which were incubated overnight at 30°C. Plates were supplemented with 20 μg ml^−1^ chloramphenicol when strains carrying pKG110-derived plasmids were assayed. When appropriate, 10 μM sodium salicylate (inducer) was added to the plates. Unless stated otherwise, 30 μg ml^−1^ cefsulodin was used.

### Aztreonam susceptibility assay.

Overnight cultures of the test strains were grown at 30°C in LB Lennox, reinoculated 1:100, and grown to an OD_578_ of 0.3 to 0.4. A 1-ml portion of each strain was centrifuged for 1 min at 13,000 × *g* and resuspended in 1 ml of 0.9% NaCl. Resuspended cells were diluted to an OD_578_ of 0.125 using 0.9% NaCl. Cells were distributed onto LB Lennox plates using a cotton swab soaked with the cell suspension. Once the plates were dried, an aztreonam MIC test strip (Liofilchem) was applied to each plate, and all the plates were incubated overnight at 30°C.

### PG synthesis assays.

[^14^C]GlcNAc-labeled lipid II (46), dansylated lipid II (47), and ATTO 550 lipid II (45, 48) were prepared as previously published. Continuous fluorescence GTase assays was performed as described elsewhere (49), using 0.5 μM PBP1A or PBP1B and a 2 μM concentration of the SPOR domain proteins, in a buffer with a final concentration of 50 mM HEPES NaOH (pH 7.5), 150 mM NaCl, 25 mM MgCl_2_ and 0.05% Triton X-100. Briefly, dansylated lipid II was added to start the reactions, and the decrease in fluorescence at 30°C was measured over time using a plate reader (excitation wavelength of 330 nm; emission wavelength of 520 nm). An endpoint GTase-TPase activity assay was performed as described elsewhere (50) using either 0.75 μM PBP1A and 1.8 μM SPOR domain proteins or 0.5 μM PBP1B and 2 μM SPOR domain proteins and a final concentration of 10 mM HEPES NaOH (pH 7.5), 150 mM NaCl, 10 mM MgCl_2_ and 0.05% Triton X-100 in the reaction buffer. Briefly, 1.2 nmol (11,000 dpm) of [^14^C]GlcNAc-labeled lipid II was dried in a glass vial using a vacuum concentrator and resuspended in 5 μl of 0.2% Triton X-100. To start the reactions, the assayed proteins were added to the resuspended lipid II and further incubated for 60 min at 37°C with shaking (800 rpm). Reactions were stopped by boiling for 5 min, and further cellosyl digestion, reduction, and analysis by HPLC were performed as described in reference 50. The following protein concentrations were used in the assays with low concentrations of PBP1A or PBP1B (0.075 μM PBP1A and 0.038 μM PBP1B) and 0.75 μM SPOR domain proteins. In samples with low PG synthase activity (with abundant unused lipid II), the total radioactivity eluted from the HPLC column (C_18_) differs between samples due to differences in peak 1, the phosphorylated disaccharide pentapeptide. Peak 1 is generated by acid hydrolysis of unused lipid II (or glycan strands ends carrying the C55-PP moiety) after the GTase-TPase reaction, because lipid II (without hydrolysis) does not elute from the C_18_ HPLC column used to separate the muropeptides. In samples with abundant unused lipid II, peak 1 varies due to differences in the efficiency of the acid hydrolysis of lipid II between samples. This effect does not impair the quantification of other peaks (PG products). Tris-Tricine SDS-PAGE was used to separate glycan strands (51), using the same protein concentrations and reaction conditions as the TPase activity experiment at low PBP1A and PBP1B concentrations but in the presence of 1 mM ampicillin to inhibit the TPase activity.

### NMR spectroscopy.

NMR data were collected in 20 mM HEPES, 300 mM NaCl, 10% D_2_O (pH 7.0), at 298 K on 1.08 mM ^13^C,^15^N-labeled DedD sample prepared in a 3-mm-diameter NMR tube. All NMR spectra for backbone, side chains, and NOE assignments were recorded on Bruker spectrometers operating at 700, 850, and 950 MHz ^1^H NMR frequencies and equipped with ^1^H,^13^C,^15^N-labeled cryoprobes.

Resonance assignments of the backbone was performed using two-dimensional (2D) ^1^H,^15^N-BEST-TROSY (BT), 3D BT-HNCANH, 3D BT-HNCO, 3D BT-HNCACO, 3D BT-HNCACB and 3D BT-HN(CO)CACB spectra. Manual side chain assignment was then achieved with conventional 2D ^1^H,^13^C-HSQC (gradient heteronuclear single quantum coherence), 3D (H)C(CCO)NH, 3D H(CCCO)NH, and 3D ^15^N-NOESY-HSQC, as well as 3D aliphatic and aromatic ^13^C-NOESY-HSQC experiments. Spectra were analyzed with CcpNmr Analysis 2.4.1.

For structural restraints, dihedral angles (phi and psi) were predicted from backbone chemical shift with the neural network program TALOS+, and distance constraints were determined after manual peak-picking and automatic assignment of the 3D NOESY-HSQC experiments reported above by Unio′10 version 2.0.2. Structures were subsequently calculated from these restraints by Aria 2.3.1, with 80 structures from runs 0 to 5, 200 for runs 6 and 7, and 600 for the last run. The 20 lowest-energy structures were further refined in water. Ramachandran analysis showed 86.1%, 13.9%, 0.0%, and 0.0% of the residues of DedD in most favored, additional allowed, generously allowed, and disallowed regions, respectively.

^1^H-^15^N NOE relaxation data were collected at 25°C on Bruker spectrometers operating at 700 MHz and equipped with ^1^H,^13^C,^15^N-labeled cryoprobes. ^1^H-^15^N NOE values were determined by the comparison of the intensities of each amide resonance with and without a 3-s saturation period and using the BEST-HETNOE sequence (52). Standard deviations were calculated from errors on peak intensities.

### Data availability.

The 20 lowest-energy structures were deposited in the PDB with accession number 6ZTG. All other data supporting the findings of this study are included in the main text and supplemental material.
