# Supplementary material for: SPOR Proteins Are Required for Functionality of Class A Penicillin-Binding Proteins in Escherichia coli
Source: mBio. 2020 Nov 3;11(6):e02796-20. doi: 10.1128/mBio.02796-20 (PMC7642682; doi:10.1128/mBio.02796-20)
Supplement: TABLE S1 [file mBio.02796-20-st001.docx]

**Table S1.** **Structural statistics for the ensemble of 20 NMR structures of *E. coli* DedD.**

| **NMR distance and dihedral constraints** | **Number / parameter** |
| --- | --- |
| Distance restraints |  |
| Total unambiguous NOE restraints | 1410 |
| Intra-residue | 550 |
| Inter-residue | 860 |
| Sequential (\|*i* – *j*\| = 1) | 389 |
| Medium-range (\|*i* – *j*\| ≤ 5) | 211 |
| Long-range (\|*i* – *j*\| > 5) | 260 |
| Total ambiguous NOE restraints | 536 |
| Total dihedral angle restraints | 248 |
| Backbone ϕ | 77 |
| Backbone ψ | 77 |
|  |  |
| **Structure calculation statistics** |  |
| Restraints violations |  |
| Distance (> 0.3 Å, > 0.5 Å) | 1.2, 3.6 |
| Dihedral (> 5°) | 2.6 |
| Average pairwise root mean square deviation (Å)^a,b^ |  |
| Backbone atoms | 0.4 ± 0.06 |
| All heavy atoms | 0.9 ± 0.07 |
| Ramachandran analysis^b^ |  |
| Residues in most favored regions (%) | 86.1 |
| Residues in additional allowed regions (%) | 13.9 |
| Residues in generously allowed regions (%) | 0.0 |
| Residues in disallowed regions (%) | 0.0 |

^a^ Pairwise r.m.s. deviation was calculated among the 20 refined structures of lowest energy.

^b^ These values were calculated on residues 142-219 (globular domain).
