# Supplementary figures and images for: SPOR Proteins Are Required for Functionality of Class A Penicillin-Binding Proteins in Escherichia coli
Source: mBio. 2020 Nov 3;11(6):e02796-20. doi: 10.1128/mBio.02796-20 (PMC7642682; doi:10.1128/mBio.02796-20)

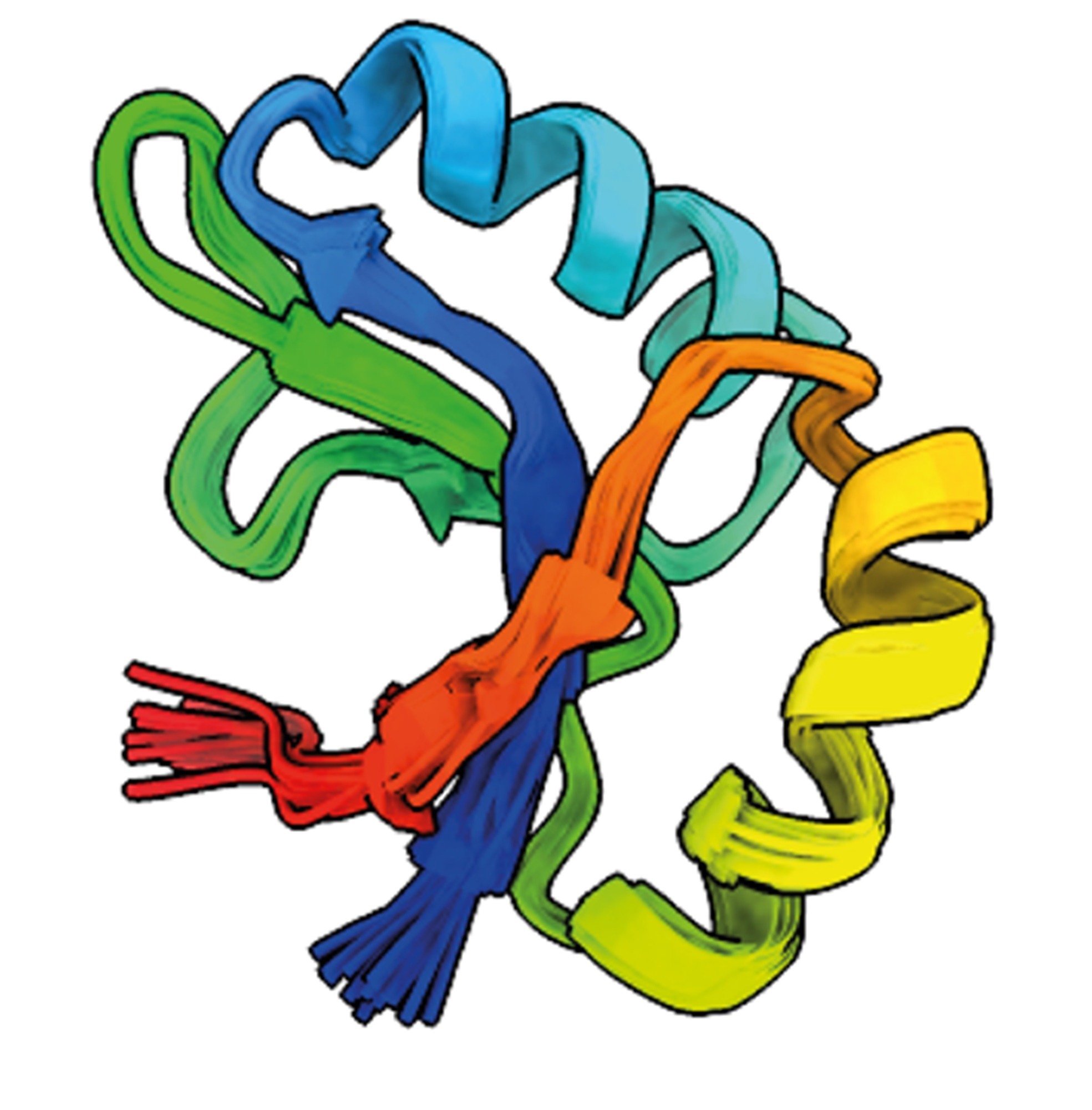

Supplement: FIG S1 [file mBio.02796-20-sf001.tif]

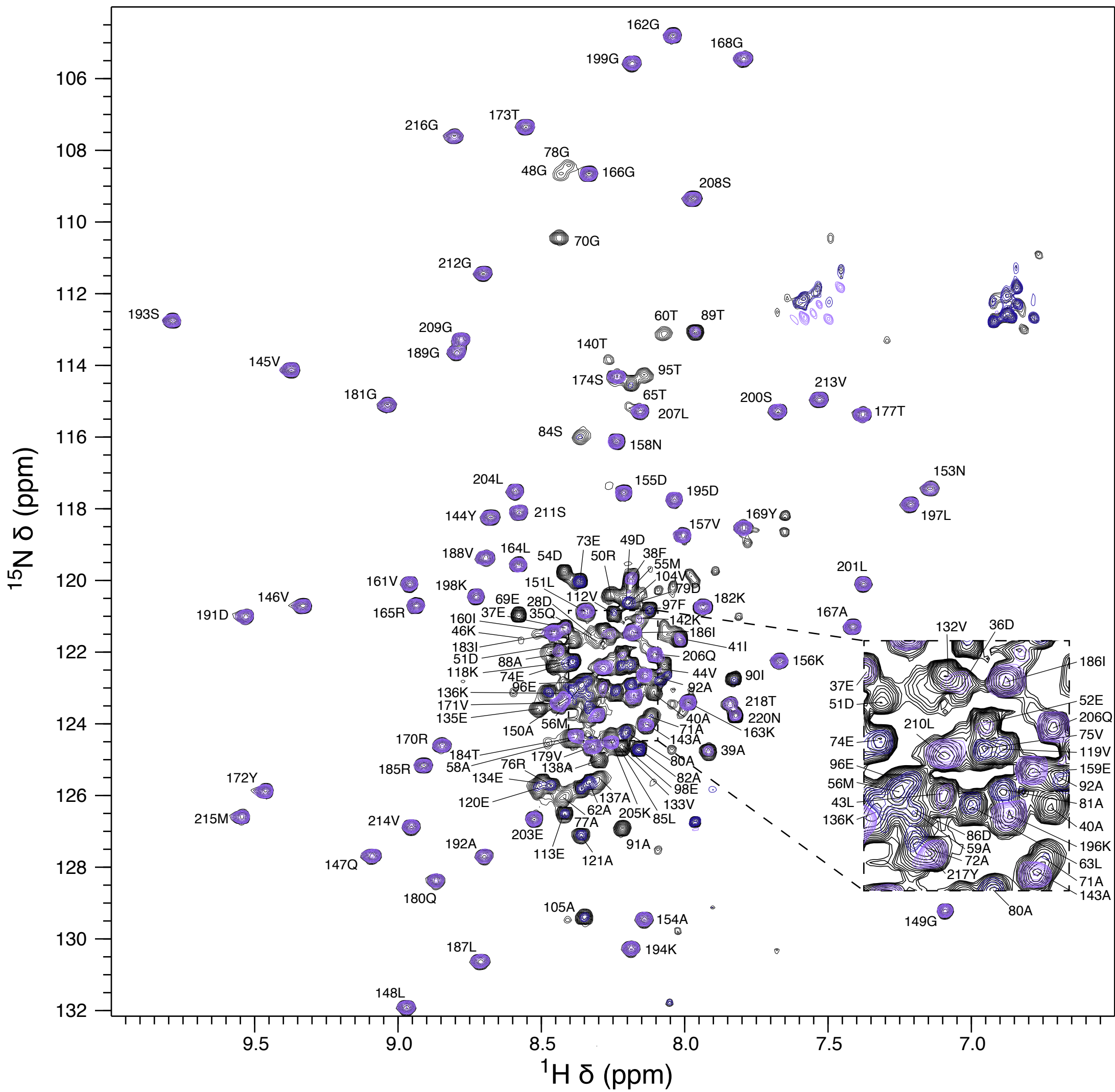

Supplement: FIG S2 [file mBio.02796-20-sf002.pdf]

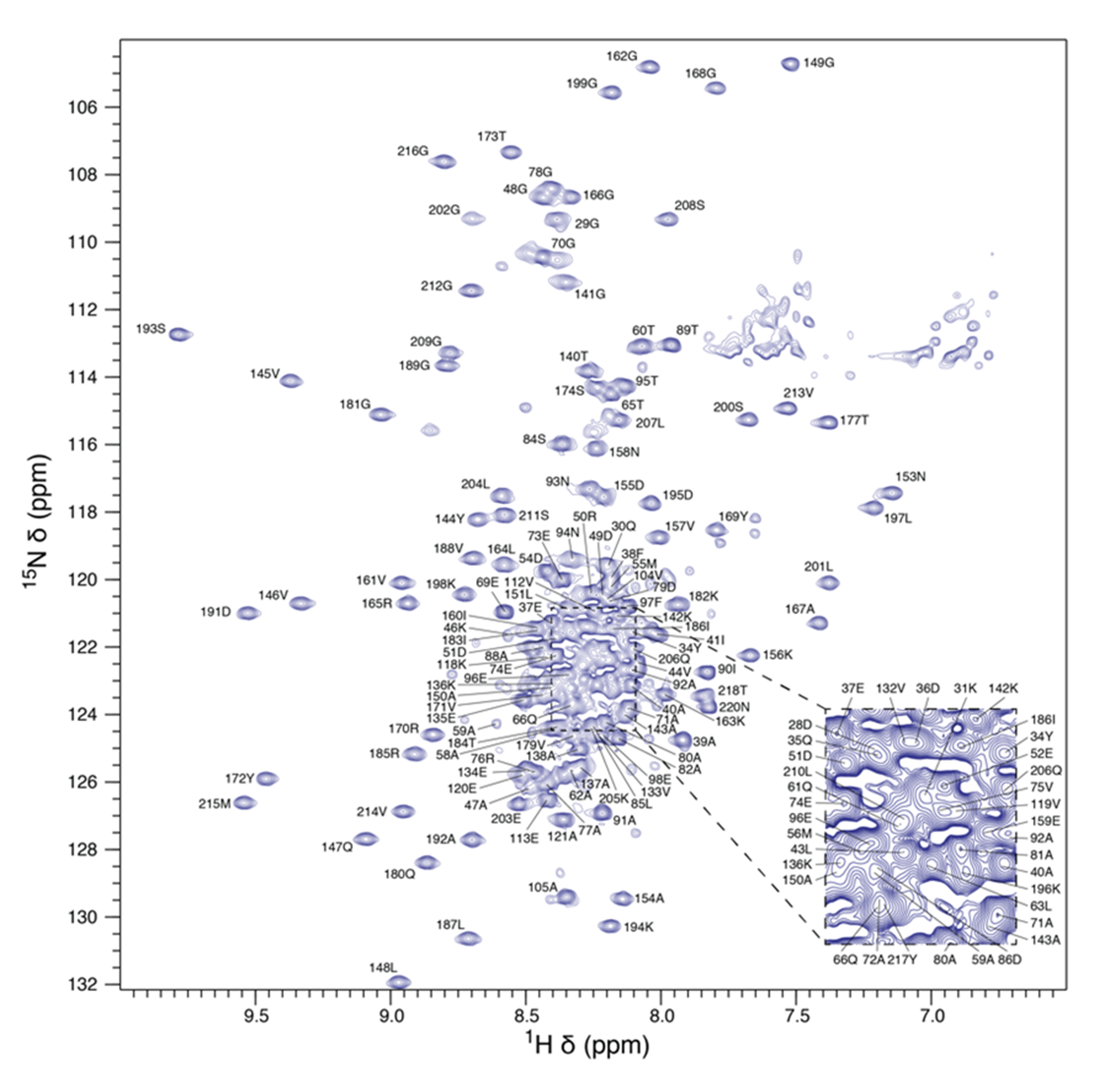

Supplement: FIG S3 [file mBio.02796-20-sf003.tif]

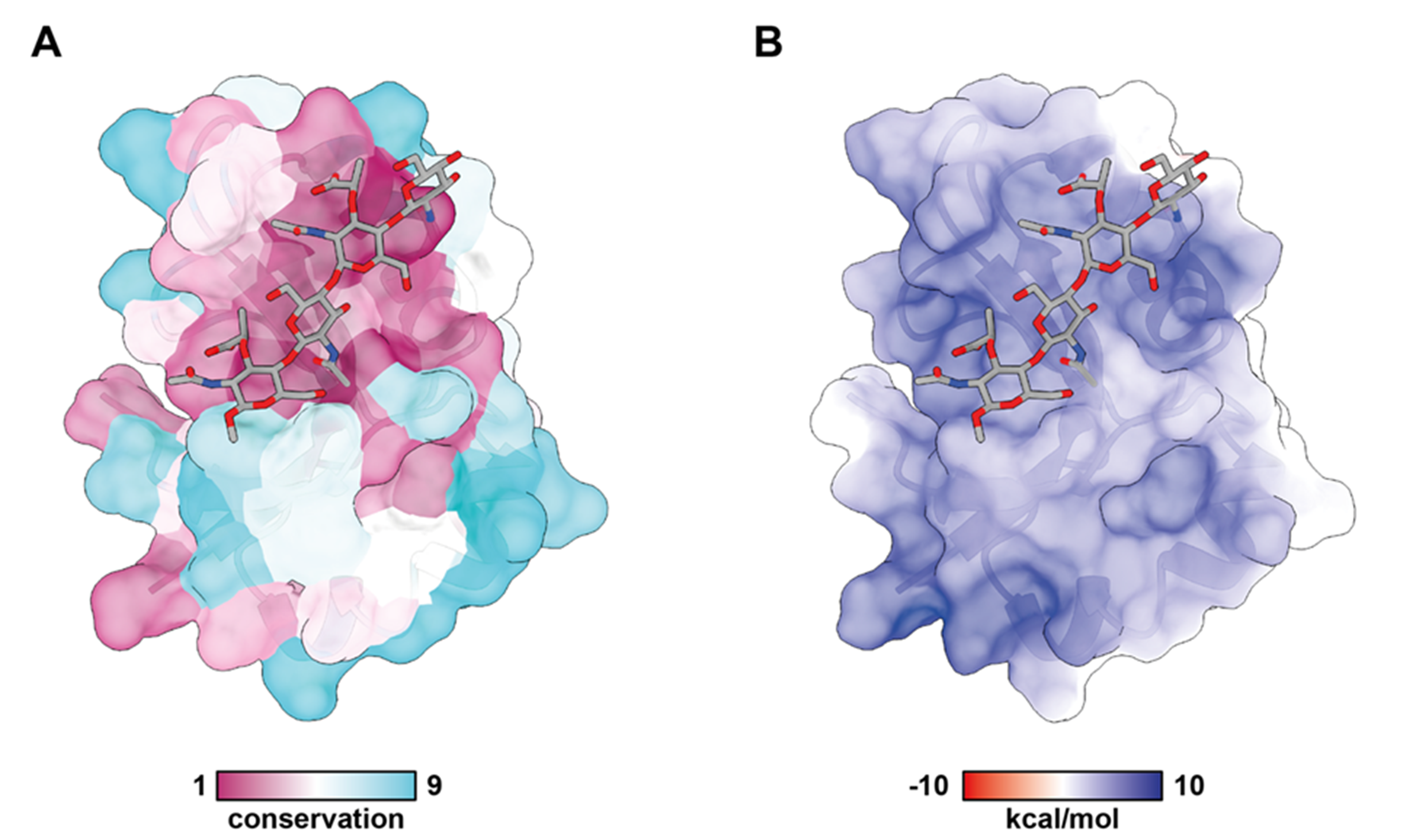

Supplement: FIG S4 [file mBio.02796-20-sf004.tif]

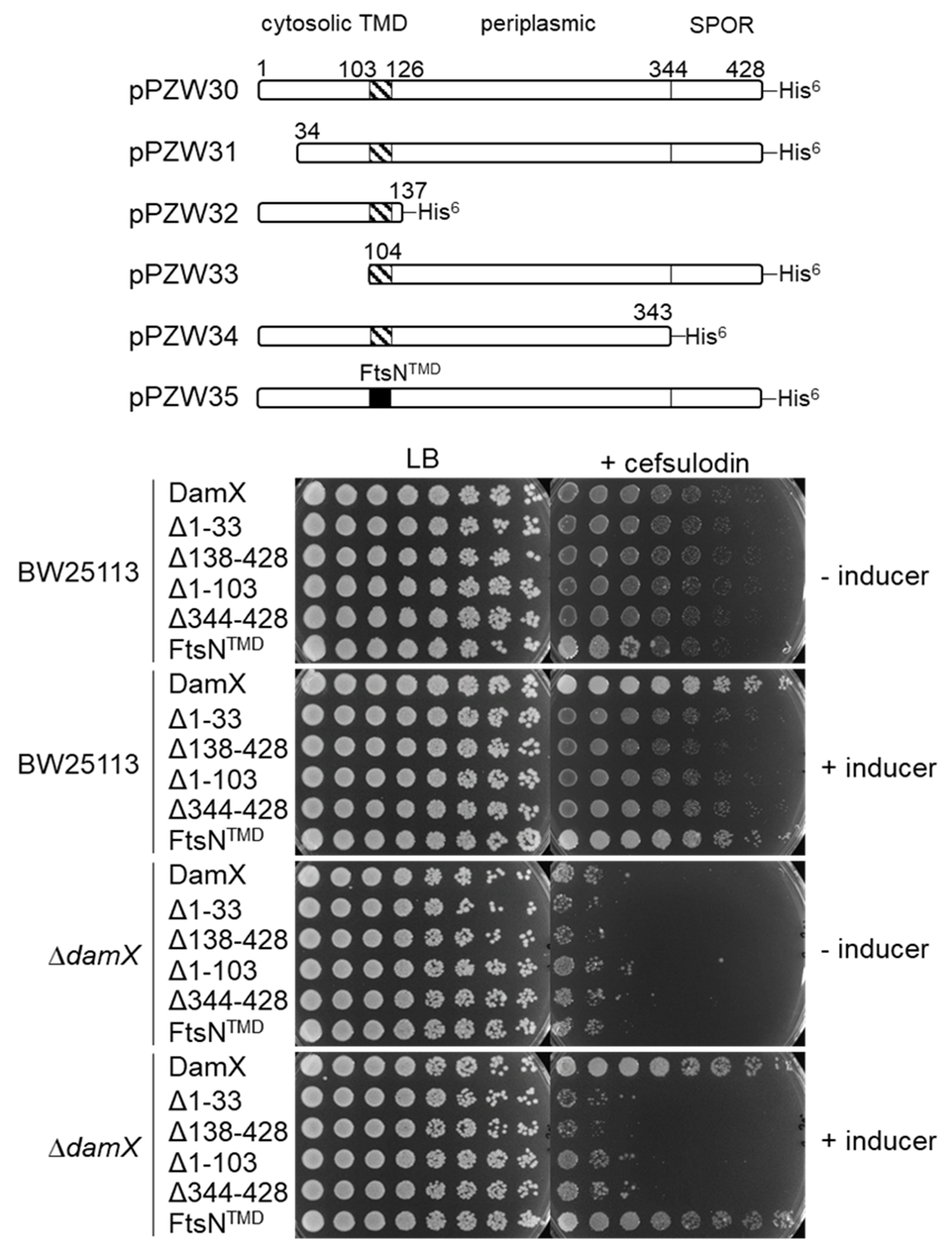

Supplement: FIG S5 [file mBio.02796-20-sf005.tif]

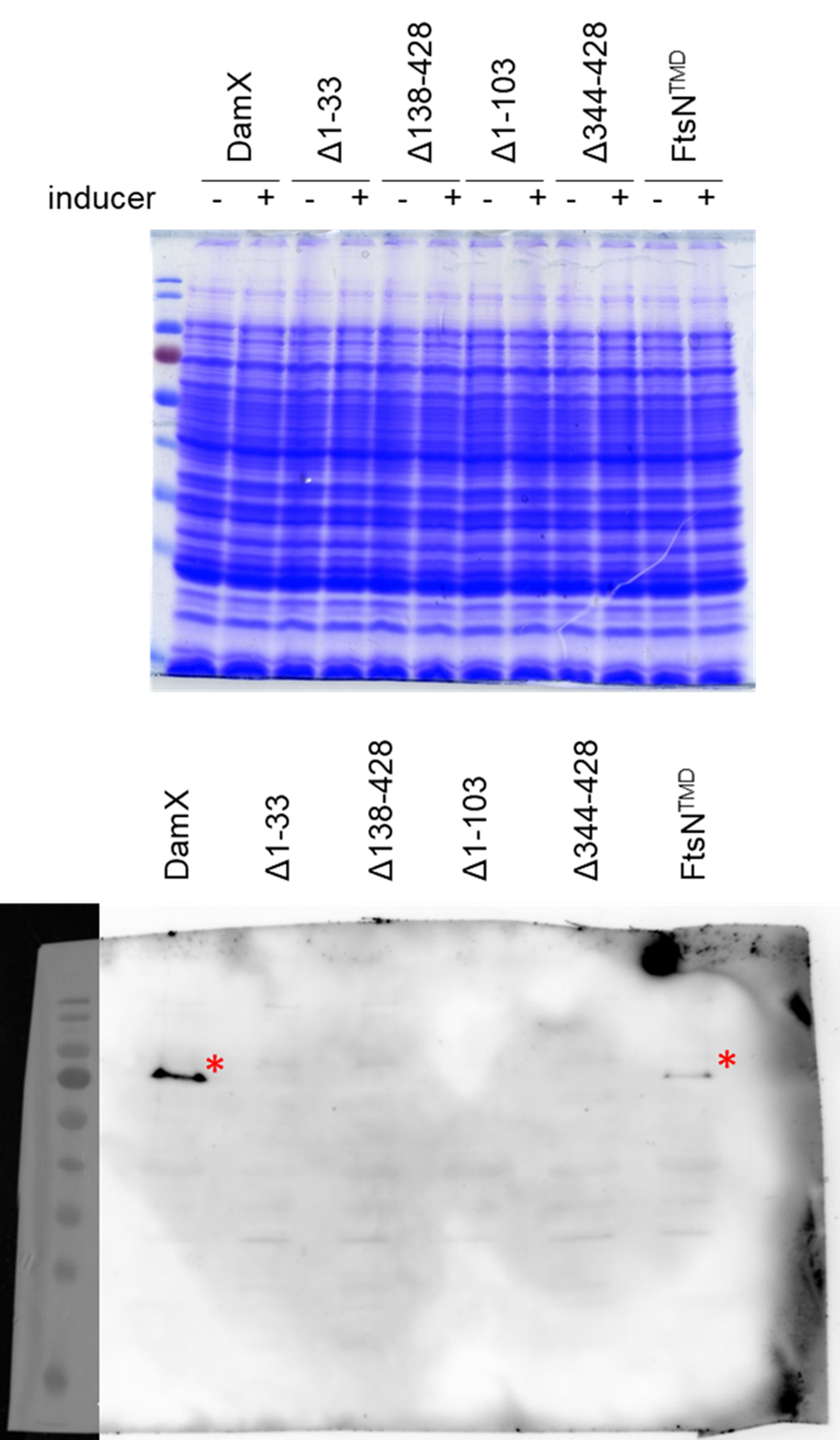

Supplement: FIG S6 [file mBio.02796-20-sf006.tif]

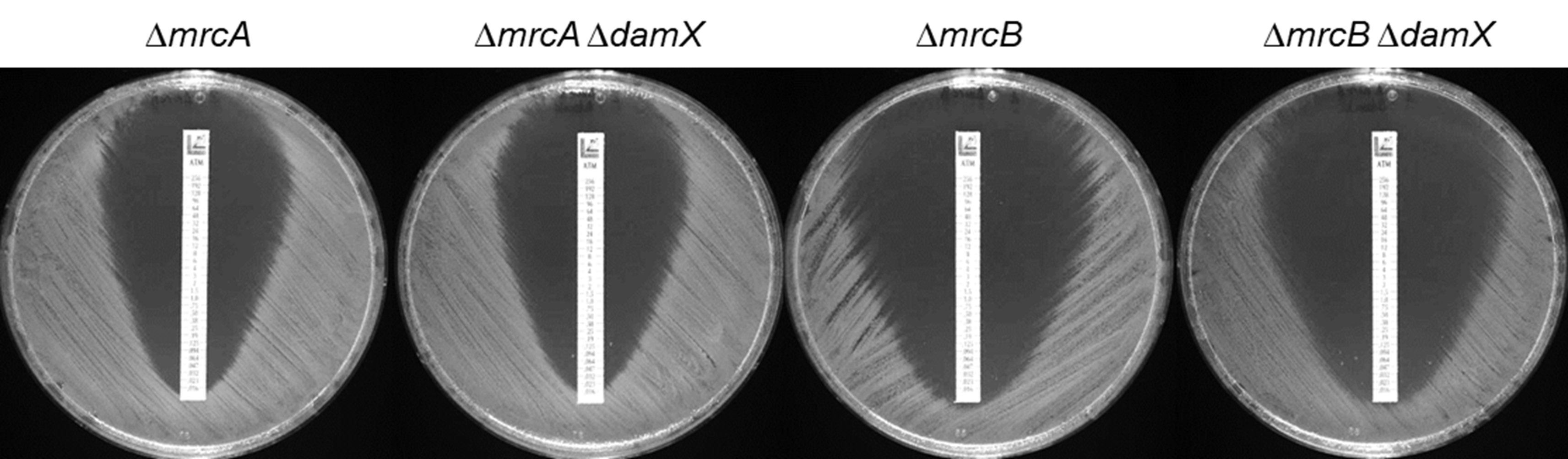

Supplement: FIG S7 [file mBio.02796-20-sf007.tif]
