# Supplementary material for: SPOR Proteins Are Required for Functionality of Class A Penicillin-Binding Proteins in Escherichia coli
Source: mBio. 2020 Nov 3;11(6):e02796-20. doi: 10.1128/mBio.02796-20 (PMC7642682; doi:10.1128/mBio.02796-20)
Supplement: TEXT S1 [file mBio.02796-20-s0001.docx]

**SUPPLEMENTAL METHODS FOR**

**SPOR proteins are required for functionality of class A Penicillin-binding proteins in *Escherichia coli***

**Manuel Pazos^1*#^, Katharina Peters^1*^, Adrien Boes^2^, Yalda Safaei^3^, Calem Kenward^3^, Nathanael A. Caveney^4^, Cedric Laguri^5^, Eefjan Breukink^6^, Natalie C. J. Strynadka^3^, Jean-Pierre Simorre^5^, Mohammed Terrak^2^, Waldemar Vollmer^1#^**

**^1^** Centre for Bacterial Cell Biology, Biosciences Institute, Newcastle University, Newcastle upon Tyne, UK.

^2^ InBioS-Centre d'Ingénierie des Protéines, Liège University, B6a, Quartier Agora, allée du six Août 11, 4000, Liège 1, Belgium.

**^3^** Biochemistry and Molecular Biology and Centre for Blood Research, The University of British Columbia, Vancouver V6T 1Z3 BC, Canada.

**^4^** Dept. of Molecular & Cellular Physiology, Standford University School of Medicine, Stanford, CA 94305, USA.

**^5^** University of Grenoble Alpes, CNRS, CEA, IBS, 38000 Grenoble, France.

**^6^** Membrane Biochemistry and Biophysics, Department of Chemistry, Faculty of Science, Utrecht University, Utrecht, The Netherlands.

^*^ Authors contributed equally to the work. Author order was determined alphabetically.

**^#^** Corresponding author; email addresses: Manuel.Pazos@newcastle.ac.uk; [Waldemar.Vollmer@newcastle.ac.uk](mailto:Waldemar.Vollmer@newcastle.ac.uk)

**SUPPLEMENTAL METHODS**

**Strains and plasmids**

*E. coli* strains and plasmids used are listed in Supplementary Table 2.

*Strains.* The strain MPW66 (BW25113 ∆*damX::frt*) was generated after removing the *frt*-flanked KanR cassette of the strain BW25113 ∆*damX::aph* by the FLP recombinase expressed from the previously introduced pCP20 plasmid (1). MPW67 (BW25113 ∆*damX::frt* ∆*mrcA::aph*) and MPW68 (BW25113 ∆*damX::frt* ∆*mrcB::aph*) were constructed using the lambda red recombineering pSIM18 plasmid (2) on strain MPW66 replacing *mrcA* with ∆*mrcA::aph* and *mrcB* with ∆*mrcB::aph*, respectively. The ∆*mrcA::aph* fragment was obtained by PCR using genomic DNA from BW25113 ∆*mrcA::aph* and the oligonucleotides Fw-up-mrcA (5’-TGA TGA TCT CCT TAT CAC CC-3’) and Rv-down-mrcA (5’-TGC AAT TTA TTG AGT TTT CAC G-3’). The ∆*mrcB::aph* fragment was obtained using genomic DNA from BW25113 ∆*mrcB::aph* and the oligonucleotides Fw-up-mrcB (5’-TCG AGC ACA AAT TTT GAG AG-3’) and Rv-down-mrcB (5’-AAC CAG ATG AAA AGA AAG GG-3’).

*Plasmids.* pETDuet constructs were generated by In-Fusion method (Takara Bio). For pETDuet-His*damX-mrcA*, *mrcA* was amplified by PCR from BW25113 genomic DNA using the oligonucleotides Fr-mrcA (5’-GGC CAC GCG ATC GCT GAC GTC ATG GTG AAG TTC GTA AAG TAT TTT TTG-3’) and Rv-mrcA (5’-CTT TAC CAG ACT CGA GTC AGA ACA ATT CCT GTG CCT CG-3’), and inserted into the MCSII of the pETDuet-1 previously digested with NdeI and XhoI. *damX* was amplified from BW25113 genomic DNA using the oligonucleotides Fr-damX (5’-ACC ACA GCC AGG ATC CAG ATG AAT TCA AAC CAG AAG ACG-3’) and Rv-damX (5’-AAG CAT TAT GCG GCC GCT TAC TTC AGA TCG GCC TGT AC-3’), and inserted into the MCSI of the pETDuet-*mrcA* previously digested with BamHI and NotI. For pETDuet-His*damX-mrcB*, *mrcB* was amplified by PCR from BW25113 genomic DNA using the oligonucleotides Fr-mrcB (5’-AAG GAG ATA TAC ATA TGC CGC GCA AAG GTA AG-3’) and Rv-mrcB (5’-CTT TAC CAG ACT CGA GTT AAT TAC TAC CAA ACA TAT CC-3’), and inserted into the MCSII of the pETDuet-1 previously digested with NdeI and XhoI. *damX* was amplified from BW25113 genomic DNA using the oligonucleotides Fr-damX (5’-ACC ACA GCC AGG ATC CAG ATG AAT TCA AAC CAG AAG ACG-3’) and Rv-damX (5’-AAG CAT TAT GCG GCC GCT TAC TTC AGA TCG GCC TGT AC-3’), and inserted into the MCSI of the pETDuet-*mrcB* previously digested with BamHI and NotI. For pETDuet-His*dedD-mrcA*, *mrcA* was amplified by PCR from BW25113 genomic DNA using the oligonucleotides Fr-mrcA (5’-GGC CAC GCG ATC GCT GAC GTC ATG GTG AAG TTC GTA AAG TAT TTT TTG-3’) and Rv-mrcA (5’-CTT TAC CAG ACT CGA GTC AGA ACA ATT CCT GTG CCT CG-3’), and inserted into the MCSII of the pETDuet-1 previously digested with NdeI and XhoI. *dedD* was amplified from BW25113 genomic DNA using the oligonucleotides Fr-dedD (5’-ACC ACA GCC AGG ATC CAG CAA GTA AGT TTC AGA ATC GGT TAG-3’) and Rv-dedD (5’-ACC ACA GCC AGG ATC CAG CAA GTA AGT TTC AGA ATC GGT TAG-3’), and inserted into the MCSI of the pETDuet-*mrcA* previously digested with BamHI and NotI. For pETDuet-His*dedD-mrcB*, *mrcB* was amplified by PCR from BW25113 genomic DNA using the oligonucleotides Fr-mrcB (5’-AAG GAG ATA TAC ATA TGC CGC GCA AAG GTA AG-3’) and Rv-mrcB (5’-CTT TAC CAG ACT CGA GTT AAT TAC TAC CAA ACA TAT CC-3’), and inserted into the MCSII of the pETDuet-1 previously digested with NdeI and XhoI. *dedD* was amplified from BW25113 genomic DNA using the oligonucleotides Fr-dedD (5’-ACC ACA GCC AGG ATC CAG CAA GTA AGT TTC AGA ATC GGT TAG-3’) and Rv-dedD (5’-ACC ACA GCC AGG ATC CAG CAA GTA AGT TTC AGA ATC GGT TAG-3’), and inserted into the MCSI of the pETDuet-*mrcB* previously digested with BamHI and NotI. pPZW23 was generated by two PCR fragments: an amplified product obtained from pET28a(+) and the oligonucleotides FwHisDamX-V (5’-CAG GCC GAT CTG AAG TAA ATC CGG CTG CTA ACA AAG CCC -3’) and RvHisDamX-V (5’-AGA ACC ACG AGG AAC TAA GCC GCT GCT GTG ATG ATG ATG-3’), and an amplified product obtained from BW25113 genomic DNA and the oligonucleotides FwHisDamX-I (5’-TTA GTT CCT CGT GGT TCT GAT GAA TTC AAA CCA GAA GAC-3’) and RvHisDamX-I (5’-CTT TGT TAG CAG CCG GAT TTA CTT CAG ATC GGC CTG TAC-3’). Same volumes of each PCR fragments were mixed, heated to 98˚C and cooled down to room temperature. The DNA mixed was digested with DpnI and transformed into DH5α competent cells. pPZW24 was obtained by the same procedure, using pET28a(+) and the oligonucleotides FwHisDedD-V (5’-GGC TAT ACG CCG AAT TAA ATC CGG CTG CTA ACA AAG CCC-3’) and RvHisDamX-V (see pPZW23), and BW25113 genomic DNA and the oligonucleotides FwHisDedD-I (5’-TTA GTT CCT CGT GGT TCT GCA AGT AAG TTT CAG AAT CGG-3’) and RvHisDedD-I (5’-CTT TGT TAG CAG CCG GAT TTA ATT CGG CGT ATA GCC C-3’). pPZW25 was obtained by the same procedure, using pET28a(+) and the oligonucleotides FwHisRlpA-V (5’-ATT ACT ACC GCG CAG TAG ATC CGG CTG CTA ACA AAG CCC-3’) and RvHisDamX-V (see pPZW23), and BW25113 genomic DNA and the oligonucleotides FwHisRlpA-I (5’-TTA GTT CCT CGT GGT TCT ACA AGC GAT GAT GGT CAG CAA C-3’) and RvHisRlpA-I (5’-CTT TGT TAG CAG CCG GAT CTA CTG CGC GGT AGT AAT AAA TG-3’). pPZW26 was obtained by the same procedure, using pET28a(+) and the oligonucleotides FwDamXHis-V (5’-TTA GTT CCT CGT GGT TCT CTC GAG CAC CAC CAC CAC CAC-3’) and RvDamXHis-V (5’-TTT GAA TTC ATC CAT CCA TGG TAT ATC TCC TTC TTA AAG-3’), and BW25113 genomic DNA and the oligonucleotides FwDamXHis-I (5’-GAA GGA GAT ATA CCA TGG ATG GAT GAA TTC AAA CCA GAA G-3’) and RvDamXHis-I (5’-AGA ACC ACG AGG AAC TAA CTT CAG ATC GGC CTG TAC CTG-3’). pPZW27 was obtained by the same procedure, using pET28a(+) and the oligonucleotides FwDamXHis-V (see pPZW26) and RvsDamXHis-V (5’-CGA GGG GGC TTT CAT CCA TGG TAT ATC TCC TTC TTA AAG-3’), and BW25113 genomic DNA and the oligonucleotides FwsDamXHis-I (5’-GAA GGA GAT ATA CCA TGG ATG AAA GCC CCC TCG ACC AC-3’) and RvDamXHis-I (see pPZW26). pPZW30 was obtained by the same procedure, using pKG110 and the oligonucleotides FwpKG110-DamXHis (5’-ACT GAG ATC CGG CTA CTA GTG GTA CCC GAG-3’) and RvDamX (5’-AAT TCA TCC ATA TGG TAC TC-3’), and pPZW26 and the oligonucleotides FwDamX (5’-TAC CAT ATG GAT GAA TTC AAA C-3’) and RvDamXHis(pKG) (5’-TCG GGT ACC ACT AGT AGC CGG ATC TCA GTG-3’). pPZW31 was obtained by the same procedure, using pKG110 and the oligonucleotides FwpKG110-DamXHis (see pPZW30) and RvDamX1-33 (5’-TTC GCC ACG CAT ATG GTA CTC-3’), and pPZW26 and the oligonucleotides FwDamX1-33 (5’-TAC CAT ATG CGT GGC GAA CCG-3’) and RvDamXHis(pKG) (see pPZW30). pPZW33 was obtained by the same procedure, using pKG110 and the oligonucleotides FwpKG110-DamXHis (see pPZW30) and RvDamX1-103 (5’-ATC ATC ATC ATA TGG TAC TC-3’), and pPZW26 and the oligonucleotides FwDamX1-103 (5’-TAC CAT ATG ATG ATG ATG GGC-3’) and RvDamXHis(pKG) (see pPZW30). pPZW37 was obtained by the same procedure, using pPZW30 and the oligonucleotides FwpKG110-dedDHis (5’-C TAT ACG CCG AAT TTA GTT CCT CGT GGT TC-3’) and RvpKG110-dedDHis (5’-CTG AAA CTT ACT TGC CAT ATG GTA CTC GTG-3’), and BW25113 genomic DNA and the oligonucleotides FwdedDHis(pKG) (5’-CAC GAG TAC CAT ATG GCA AGT AAG TTT CAG-3’) and RvdedDHis(pKG) (5’-ACC ACG AGG AAC TAA ATT CGG CGT ATA GCC-3’). pPZW45 was obtained by the same procedure, using pPZW30 and the oligonucleotides FwpKG110-rlpAHis (5’-T ACT ACC GCG CAG TTA GTT CCT CGT GGT TC-3’) and RvpKG110-rlpAHis (5’-GAG CCA CTG CTT ACG CAT ATG GTA CTC GTG-3’), and BW25113 genomic DNA and the oligonucleotides FwrlpAHis(pKG) (5’-CAC GAG TAC CAT ATG CGT AAG CAG TGG CTC-3’) and RvrlpAHis(pKG) (5’-ACC ACG AGG AAC TAA CTG CGC GGT AGT AAT-3’). pPZW32, pPZW34 and pPZW35 were obtained using a Q5 site-directed mutagenesis kit (NEB), pPZW30 as DNA template and the oligonucleotides Fw damXHisΔSPOR (5’-TTA GTT CCT CGT GGT TCT C-3’) and RvdamX1-137 (5’-AGA CGC GGT TTG ATC GCT-3’), Fw damXHisΔSPOR and Rv damXHisΔSPOR (5’-TGC CGA TTT CAA CGA ACC-3’), and Fw FtsNTMDDamX (5’-TGT GAC CTT TAT CGG TGG TCT GTA CTT CAT TAA AGC CCC CTC GAC CAC T-3’) and Rv FtsNTMDDamX (5’-AGA ACG GCG GCA GCA ATA GCG ACC ATA GCG GGA TAC TGA CGA GAA GCG GG-3’) respectively. pYS001 was obtained through restriction free cloning of *E. coli* DedD residues 28-220 into a pET28a(+) vector. Fw YS001 primer (5’- GCG GCC TGG TGC CGC GCG GCA GCC ATA TGG ACG GGC AGA AAA AAC ATT ATC AGG ATG-3’) and Rv YS001 primer (5’- CGG GCT TTG TTA GCA GCC GGA TCT CAT TAT TAA TTC GGC GTA TAG CCC ATT ACC ACG-3’). Constructs were confirmed by DNA sequencing the genes of interest.

**SUPPLEMENTAL REFERENCES**

1. Cherepanov PP, Wackernagel W. 1995. Gene disruption in *Escherichia coli*: TcR and KmR cassettes with the option of Flp-catalyzed excision of the antibiotic-resistance determinant. Gene 158:9-14.

2. Datta S, Costantino N, Court DL. 2006. A set of recombineering plasmids for gram-negative bacteria. Gene 379:109-15.
