# Supplementary material for: SPOR Proteins Are Required for Functionality of Class A Penicillin-Binding Proteins in Escherichia coli
Source: mBio. 2020 Nov 3;11(6):e02796-20. doi: 10.1128/mBio.02796-20 (PMC7642682; doi:10.1128/mBio.02796-20)
Supplement: TABLE S2 [file mBio.02796-20-st002.docx]

**Table S2. Strains and plasmids.**

| **Strain or plasmid** | **Genotype / properties** | **Source or reference** |
| --- | --- | --- |
| **Strain** |  |  |
| BL21(DE3) | Expression strain | Novagen |
| BW25113 | Wild type strain | (1) |
| BW25113 ∆*damX* | BW25113 ∆*damX::aph* | (2) |
| BW25113 ∆*dedD* | BW25113 ∆*dedD::aph* | (2) |
| BW25113 ∆*mrcA* | BW25113 ∆*mrcA::aph* | (2) |
| BW25113 ∆*mrcB* | BW25113 ∆*mrcB::aph* | (2) |
| BW25113 ∆*rlpA* | BW25113 ∆*rlpA::aph* | (2) |
| C43(DE3) | Membrane overproduction strain | (3) |
| LOBSTR | Expression strain | Kerafast |
| MPW66 | BW25113 ∆*damX::frt* | This work |
| MPW67 | BW25113 ∆*damX::frt* ∆*mrcA::aph* | This work |
| MPW68 | BW25113 ∆*damX::frt* ∆*mrcB::aph* | This work |
| **Plasmid** |  |  |
| pCP20 | FLP overproduction, Cm^R^, Ap^R^ | (4) |
| pDML924 | pET28a(+) encoding His-PBP1Bγ | (5) |
| pET28a(+) | Overexpression of gene of interest, Kan^R^ | Novagen |
| pETDuet-1 | Co-expression of two genes of interes, Ap^R^ | Novagen |
| pETDuet-His*damX-mrcA* | pETDuet-1 encoding His-DamX and PBP1A | This work |
| pETDuet-His*damX-mrcB* | pETDuet-1 encoding His-DamX and PBP1B | This work |
| pETDuet-His*dedD-mrcA* | pETDuet-1 encoding His-DedD and PBP1A | This work |
| pETDuet-His*dedD-mrcB* | pETDuet-1 encoding His-DedD and PBP1B | This work |
| pFE42 | pHis17 encoding FtsN-His | (6) |
| pKG110 | pLC112 backbone, Cam^R^, Pnah | J. S. Parkinson |
| pPZW23 | pET28a(+) encoding His-DamX | This work |
| pPZW24 | pET28a(+) encoding His-DedD | This work |
| pPZW25 | pET28a(+) encoding His-RlpA∆1-18 | This work |
| pPZW26 | pET28a(+) encoding DamX-His | This work |
| pPZW27 | pET28a(+) encoding DamX∆1-124-His | This work |
| pPZW30 | pKG110 encoding DamX-His | This work |
| pPZW31 | pKG110 encoding DamX∆1-33-His | This work |
| pPZW32 | pKG110 encoding DamX∆138-428-His | This work |
| pPZW33 | pKG110 encoding DamX∆1-103-His | This work |
| pPZW34 | pKG110 encoding DamX∆344-428-His | This work |
| pPZW35 | pKG110 encoding DamX-His (DamX104-124::FtsN34-54) | This work |
| pPZW37 | pKG110 encoding DedD-His | This work |
| pPZW45 | pKG110 encoding RlpA-His | This work |
| pSIM18 | Lambda red recombineering | (7) |
| pTK1Ahis | pET28a(+) encoding His-PBP1A | (8) |
| pYS001 | pET28a(+) encoding His-solDedD (28-220) | This work |

**Supplemental References**

1. Datsenko KA, Wanner BL. 2000. One-step inactivation of chromosomal genes in *Escherichia coli* K-12 using PCR products. Proc Natl Acad Sci U S A 97:6640-5.

2. Baba T, Ara T, Hasegawa M, Takai Y, Okumura Y, Baba M, Datsenko KA, Tomita M, Wanner BL, Mori H. 2006. Construction of *Escherichia coli* K-12 in-frame, single-gene knockout mutants: the Keio collection. Mol Syst Biol 2:2006 0008.

3. Miroux B, Walker JE. 1996. Over-production of proteins in *Escherichia coli*: mutant hosts that allow synthesis of some membrane proteins and globular proteins at high levels. J Mol Biol 260:289-98.

4. Cherepanov PP, Wackernagel W. 1995. Gene disruption in *Escherichia coli*: TcR and KmR cassettes with the option of Flp-catalyzed excision of the antibiotic-resistance determinant. Gene 158:9-14.

5. Terrak M, Ghosh TK, van Heijenoort J, Van Beeumen J, Lampilas M, Aszodi J, Ayala JA, Ghuysen JM, Nguyen-Disteche M. 1999. The catalytic, glycosyl transferase and acyl transferase modules of the cell wall peptidoglycan-polymerizing penicillin-binding protein 1b of *Escherichia coli*. Mol Microbiol 34:350-64.

6. Yang JC, Van Den Ent F, Neuhaus D, Brevier J, Lowe J. 2004. Solution structure and domain architecture of the divisome protein FtsN. Mol Microbiol 52:651-60.

7. Datta S, Costantino N, Court DL. 2006. A set of recombineering plasmids for gram-negative bacteria. Gene 379:109-15.

8. Born P, Breukink E, Vollmer W. 2006. In vitro synthesis of cross-linked murein and its attachment to sacculi by PBP1A from *Escherichia coli*. J Biol Chem 281:26985-93.
